# Thioredoxin-1 decreases alpha-synuclein induced by MPTP through promoting autophagy-lysosome pathway

**DOI:** 10.1038/s41420-024-01848-0

**Published:** 2024-02-22

**Authors:** Rou Gu, Liping Bai, Fang Yan, Se Zhang, Xianwen Zhang, Ruhua Deng, Xiansi Zeng, Bo Sun, Xiaomei Hu, Ye Li, Jie Bai

**Affiliations:** 1 Faculty of Life Science and Technology, Kunming University of Science and Technology, Kunming, China; 2 Laboratory of Molecular Neurobiology, Medical School, Kunming University of Science and Technology, Kunming, China

**Keywords:** Macroautophagy, Parkinson's disease

## Abstract

Parkinson’s disease (PD) is characterized by the formation of Lewy body in dopaminergic neurons in the substantia nigra pars compacta (SNpc). Alpha-synuclein (α-syn) is a major component of Lewy body. Autophagy eliminates damaged organelles and abnormal aggregated proteins. Thioredoxin-1 (Trx-1) is a redox regulating protein and plays roles in protecting dopaminergic neurons against neurotoxicity induced by 1-methyl-4-phenyl-1,2,3,6-tetrahydropyridine (MPTP). However, the relationship between Trx-1 and α-syn in PD is still unknown. In the present study, the movement disorder and dopaminergic neurotoxicity in MPTP-treated mice were improved by Trx-1 overexpression and were aggravated by Trx-1 knockdown in the SNpc in mice. The expression of α-syn was increased in the SNpc of MPTP-treated mice, which was inhibited by Trx-1 overexpression and was exacerbated in Trx-1 knockdown mice. Autophagosomes was increased under electron microscope after MPTP treatment, which were recovered in Trx-1 overexpressing mice and were further increased in Trx-1 knockdown in the SNpc in mice. The expressions of phosphatase and tensin homolog deleted on chromosome ten (PTEN)-induced putative kinase 1 (PINK1), Parkin, LC3 II and p62 were increased by MPTP, which were blocked in Trx-1 overexpressing mice and were further increased in Trx-1 knockdown mice. Cathepsin D was decreased by MPTP, which was restored in Trx-1 overexpressing mice and was further decreased in Trx-1 knockdown mice. The mRFP-GFP-LC3 green fluorescent dots were increased by 1-methyl-4-phenylpyridinium (MPP^+^) and further increased in Trx-1 siRNA transfected PC12 cells, while mRFP-GFP-LC3 red fluorescent dots were increased in Trx-1 overexpressing cells. These results indicate that Trx-1 may eliminate α-syn in PD mice through potentiating autophagy-lysosome pathway.

## Introduction

Parkinson’s disease (PD) is a neurodegenerative disease characterized by movement disorder. The pathogenesis of PD is the loss of dopaminergic neurons in the substantia nigra pars compacta (SNpc), and abnormal accumulation of alpha-synuclein (α-syn) which is the main constituent of Lewy bodies. The mechanism on PD is related to autophagy and lysosome dysfunction [1–3].

Autophagy is a cellular process, which degrades damaged cytoplasmic materials, such as proteins and organelles in lysosomes, and exerts the protection function against various stresses [4]. When autophagic-lysosomal degradative process is impaired, abnormally aggregated α-syn fails to be eliminated timely. In turn, misfolded α-syn interferes the degradation pathway, leading to toxic substances and damaged organelles accumulation [5]. Therefore, the maintenance of autophagic and lysosomal functions is critical in PD.

Phosphatase and tensin homolog deleted on chromosome ten (PTEN)-induced putative kinase 1 (PINK1) is a kinase, which stabilizes on the surface of damaged mitochondria. As the upstream of Parkin, PINK1 phosphorylates both Parkin and ubiquitin, then promotes the recruitment of mitophagy receptors [6]. Parkin ubiquitinates proteins on outer mitochondrial membrane to trigger selective autophagy [7]. PINK1 and Parkin have been proved to functionally interact with α-syn, and the expression of the two proteins is beneficial to α-syn degradation [8, 9].

Microtubule-associated protein 1 light chain 3 (LC3) is engaged in the formation of autophagosomes. LC3 is cleaved to form LC3 I firstly, and then LC3 I is conjugated to phosphatidylethanolamine by autophagy proteins to form LC3 II, and LC3 II combines with autophagosome membranes [4]. As receptor protein, p62 binds to aggregated proteins selectively and combines with LC3 family members on autophagosomes through LC3 interacting region motifs [10]. In addition, lysosomal enzymes are indispensable in autophagy process.

Thioredoxin-1 (Trx-1) is a redox regulating protein and plays roles in regulating cellular redox processes and neuroprotection [11]. Our previous studies showed that the expression of Trx-1 was reduced by methyl-4-phenyl-1,2,3,6-tetrahydropyridine (MPTP)/1-methyl-4-phenylpyridinium (MPP^+^). Overexpression of Trx-1 protected dopaminergic neurons from MPTP toxicity in the SNpc of mice by suppressing the endoplasmic reticulum stress [12]. Recently, we found that Trx-1 played an important role in regulating ferroptosis in PD mice [13]. However, whether Trx-1 regulates the process of autophagy and α-syn accumulation in PD has not been addressed.

In the present study, we explored whether Trx-1 eliminated α-syn induced by MPTP through enhancing autophagy-lysosome pathway in PD mice.

## Results

### Trx-1 ameliorated MPTP-induced motor impairment of mice

**Fig. 1:**
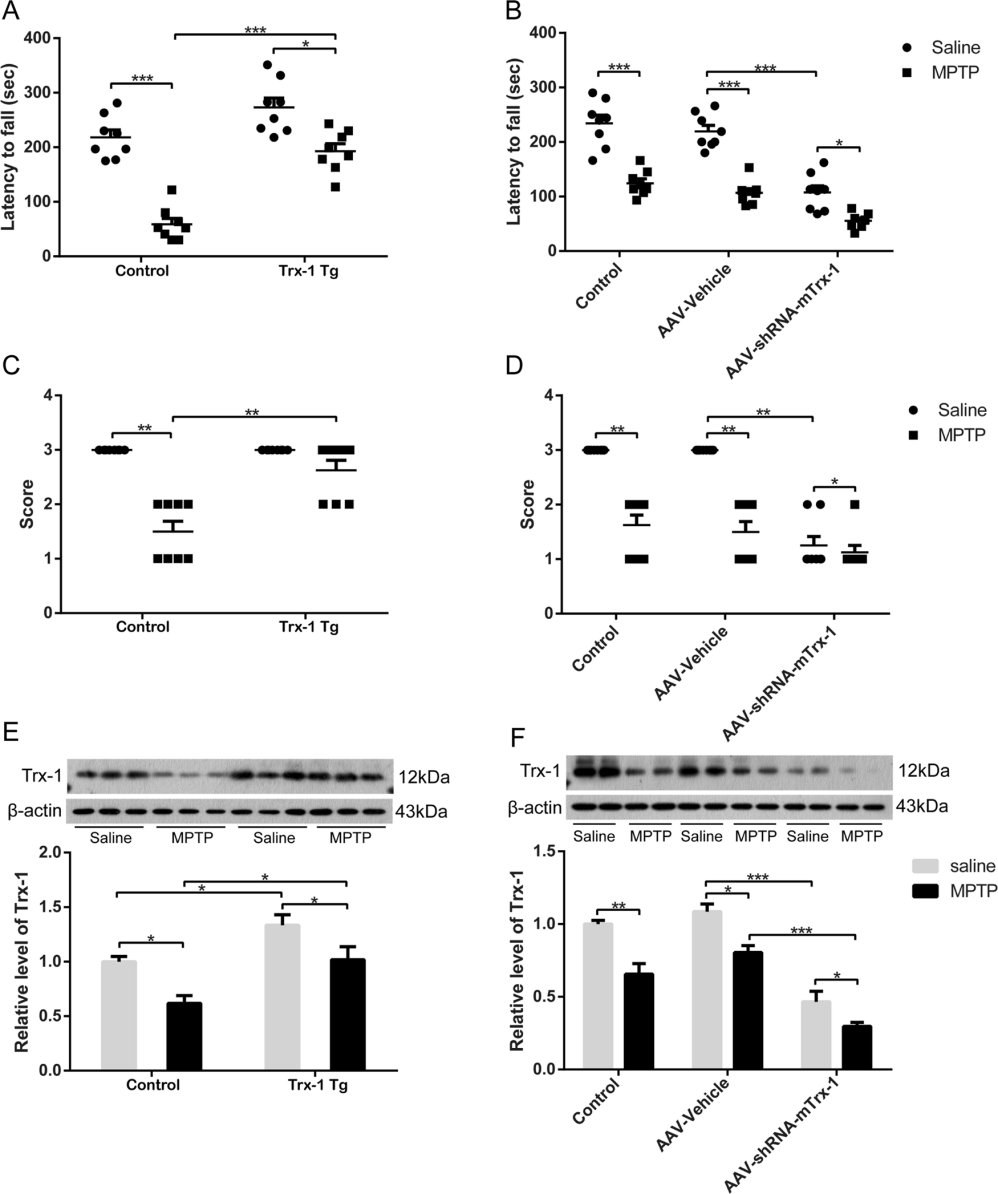
Trx-1 effects on MPTP-induced motor impairment of mice. Mice were treated with MPTP (30 mg/kg, once a day, intraperitoneally). Rotarod test, shorter latency in the MPTP group was recovered in Trx-1 overexpressing transgenic mice (Tg) + MPTP group **A** and was aggravated in Trx-1 knockdown in the SNpc in mice + MPTP group **B**. Grip strength test, lower score in the MPTP group was recovered in Trx-1 overexpressing transgenic (Tg) mice + MPTP group **C** and was aggravated in Trx-1 knockdown in the SNpc in mice + MPTP group **D**. Each bar represents the mean ± SEM (n = 8). *P < 0.05, **P < 0.01, ***P < 0.001, statistically significant. The expression of Trx-1 in the SNpc in mice treated with MPTP was detected by Western blot analysis **E**, **F**. Each bar represents the mean ± SEM (n = 6). *P < 0.05, **P < 0.01, ***P < 0.001, statistically significant.

MPTP was used to establish PD models in vivo. To determine the effect of Trx-1 on movement disorder in MPTP-treated mice, a series of behaviors were assessed. The mice treated with MPTP showed poor ability to the rotating stick and the movement time on the rotarod was significantly shortened, which were rescued by Trx-1 overexpression (Fig. 1A; two-way ANOVA: MPTP: F_(1,28)_ = 45.86, *P* < 0.001；genotype: F_(1,28)_ = 73.57, *P* < 0.001; interaction: F_(1,28)_ = 8.050, *P* < 0.05) and aggravated by Trx-1 knockdown in the SNpc (Fig. 1B; two-way ANOVA: MPTP: F_(2,42)_ = 51.00, *P* < 0.001；genotype: F_(1,42)_ = 116.8, *P* < 0.001; interaction: F_(2,42)_ = 5.437, *P* < 0.05).

The wire grasp activity of the hind paw of mice was decreased by MPTP, which was recovered by Trx-1 overexpression (Fig. 1C; two-way ANOVA: MPTP: F_(1,28)_ = 18.29, *P* < 0.01；genotype: F_(1,28)_ = 50.81, *P* < 0.001; interaction: F_(1,28)_ = 18.29, *P* < 0.01). and further enhanced by Trx-1 knockdown in the SNpc (Fig. 1D; two-way ANOVA: MPTP: F_(2,42)_ = 42.98, *P* < 0.001；genotype: F_(1,42)_ = 80.64, *P* < 0.001; interaction: F_(2,42)_ = 15.54, *P* < 0.001).

To examine whether Trx-1 expression is related the motor deficits induced by MPTP, Trx-1 expression was detected by Western blot analysis. Trx-1 expression was decreased by MPTP, which was restored in Trx-1 overexpressing mice (Fig. 1E; two-way ANOVA: MPTP: F_(1,20)_ = 15.5, *P* < 0.01；genotype: F_(1,20)_ = 17.11, *P* < 0.01; interaction: F_(1,20)_ = 0.1390, *P* > 0.05), and was further decreased in mice of Trx-1 knockdown in the SNpc (Fig. 1F; two-way ANOVA: MPTP: F_(2,18)_ = 62.76, *P* < 0.001；genotype: F_(1,18)_ = 37.42, *P* < 0.001; interaction: F_(2,18)_ = 1.397, *P* > 0.05).

Taken together, Trx-1 overexpression ameliorated motor deficits induced by MPTP, and Trx-1 knockdown aggravated it, indicating that Trx-1 is involved in movement disorder induced by MPTP in mice.

### Trx-1 resisted the dopaminergic neurotoxicity in MPTP-treated mice

**Fig. 2:**
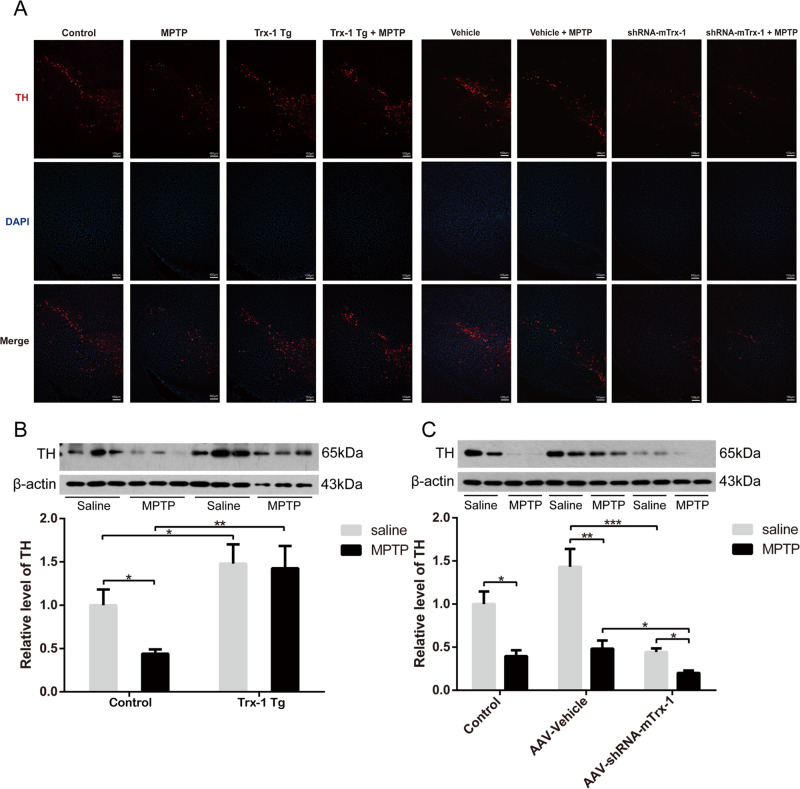
Effects of Trx-1 on expression of TH in the SNpc in MPTP-treated mice. The TH expression in the SNpc was detected by immunofluorescence staining analysis in Trx-1 overexpressing mice and Trx-1 knockdown in the SNpc **A**. The MPTP-decreased TH expression was restored in Trx-1 overexpressing transgenic (Tg) mice **B** and was more decreased in Trx-1 knockdown in the SNpc in mice **C**. Each bar represents the mean ± SEM (n = 6). *P < 0.05, **P < 0.01, ***P < 0.001, statistically significant.

Tyrosine hydroxylase (TH) is a key enzyme for dopamine biosynthesis. Thus, TH is generally regarded as a biomarker for dopaminergic neurons. TH expression in the SNpc was detected by immunofluorescence staining. After MPTP treatment, the number of TH-positive neurons was significantly reduced in the SNpc, which was restored in Trx-1 overexpressing mice, and was further decreased in Trx-1 knockdown mice (Fig. 2A). Western blot analysis on TH expression showed similar result with Trx-1 expression. TH expression in the SNpc was decreased by MPTP, which was restored in Trx-1 overexpressing mice (Fig. 2B; two-way ANOVA: MPTP: F_(1,20)_ = 12.493, *P* < 0.05；genotype: F_(1,20)_ = 14.18, *P* < 0.05; interaction: F_(1,20)_ = 1.648, *P* > 0.05), and was further decreased in mice of Trx-1 knockdown in the SNpc (Fig. 2C; two-way ANOVA: MPTP: F_(2,18)_ = 15.31, *P* < 0.01；genotype: F_(1,18)_ = 40.29, *P* < 0.001; interaction: F_(2,18)_ = 4.676, *P* < 0.05). These results suggest that Trx-1 plays a role in resisting the dopaminergic neurotoxicity of MPTP in the SNpc.

### Trx-1 reduced the expression of α-syn in MPTP-treated mice

**Fig. 3:**
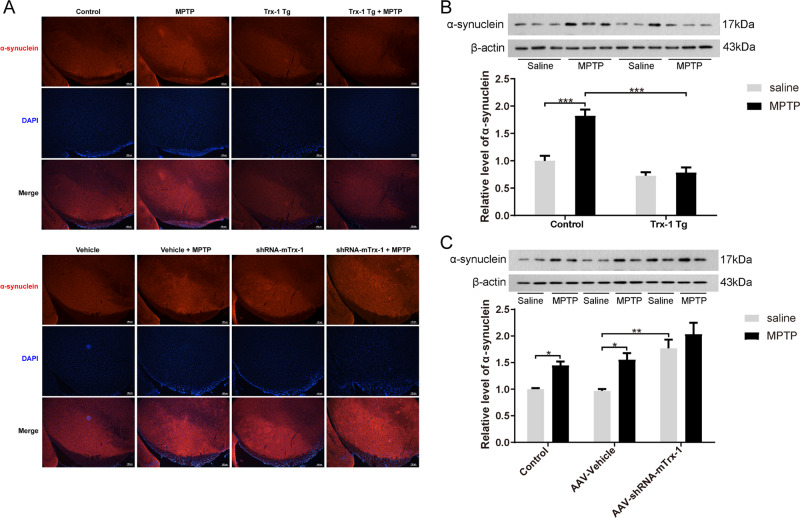
Effects of Trx-1 on the clearance of α-syn in the SNpc in MPTP- treated mice. The α-syn expression in the SNpc was detected by immunofluorescence staining analysis in Trx-1 overexpressing mice and Trx-1 knockdown in the SNpc **A**. The MPTP promoted α-syn expression, which was repressed in Trx-1 overexpressing transgenic (Tg) mice **B** and was more increased in Trx-1 knockdown in the SNpc in mice **C**. Each bar represents the mean ± SEM (*n* = 6). *P < 0.05, **P < 0.01, ***P < 0.001, statistically significant.

Expression of α-syn usually is increased by MPTP. Thus, the expression of α-syn in the SNpc was detected by immunofluorescence staining and Western Blot analysis. As expected, α-syn expression was increased by MPTP in the SNpc compared to WT mice, which was inhibited in Trx-1 overexpressing mice, and was further exacerbated in Trx-1 knockdown mice (Fig. 3A). The similar results were also obtained by Western Blot analysis (Fig. 3B; two-way ANOVA: MPTP: F_(1,20)_ = 33.8, *P* < 0.001；genotype: F_(1,20)_ = 10.9, *P* < 0.01; interaction: F_(1,20)_ = 25.9, *P* < 0.001; Fig. 3C; two-way ANOVA: MPTP: F_(2,18)_ = 18.5, *P* < 0.001；genotype: F_(2,18)_ = 18.1, *P* < 0.001; interaction: F_(1,18)_ = 0.833, *P* > 0.05). To further check whether α-syn was altered transcriptionally, the mRNA level of α-syn was detected by RT-qPCR. The mRNA level of α-syn was increased by MPTP, which was inhibited in Trx-1 overexpressing mice (Fig. S1A, two-way ANOVA: MPTP: F_(1,20)_ = 21.34, *P* < 0.001; genotype: F_(1,20)_ = 26.86, *P* < 0.001; interaction F_(1,20)_ = 8.019, *P* < 0.05), and was further increased in Trx-1 knockdown mice (Fig. S1B, two-way ANOVA: MPTP: F_(1,20)_ = 19.02, *P* < 0.001; genotype: F_(1,20)_ = 31.36, *P* < 0.001; interaction: F_(1,20)_ = 0.0001243, *P* > 0.05). These results demonstrated that Trx-1 was involved in the expression of α-syn increased by MPTP.

### Effect of Trx-1 on autophagy in MPTP-treated mice

**Fig. 4:**
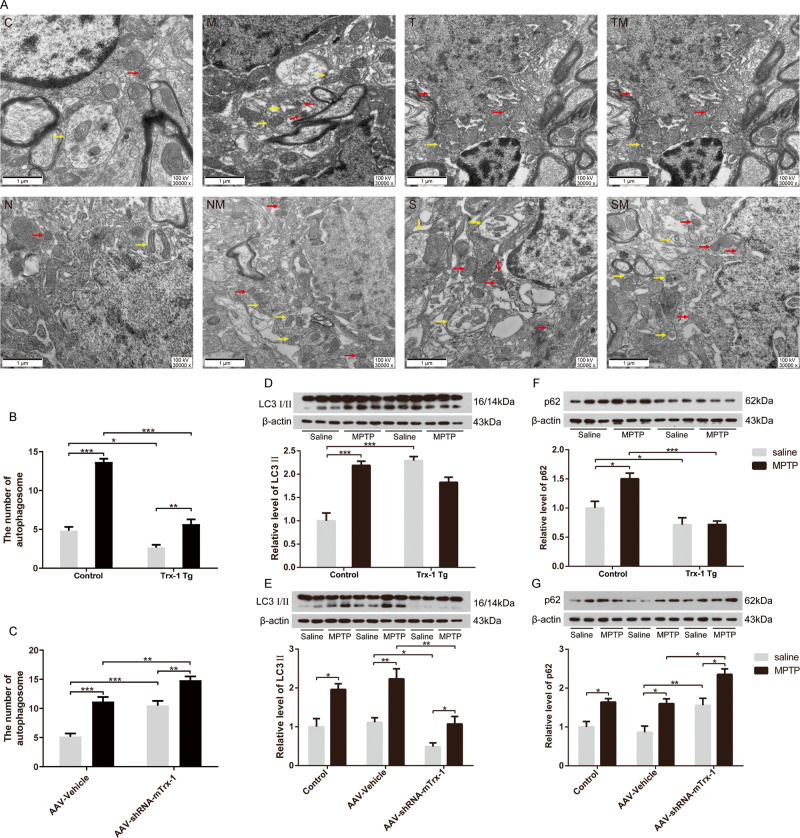
Effects of Trx-1 on autophagy in MPTP- treated mice. There are some normal morphology mitochondria, round or oval with fewer autophagosomes in control mice (C). The increase in number of changed morphology mitochondria characterized by mitochondrial crest structure disappeared. The mitochondria were significantly increased with a double membrane structure and a large number of autophagosomes in mice treated with MPTP (M). There are normal morphology mitochondria, round or oval with fewer autophagosomes in Trx-1 overexpressing mice (T). The changed morphology mitochondria and mitochondria with a double membrane structure were fewer in Trx-1 overexpressing mice treated with MPTP (TM). There are some normal morphology mitochondria, round or oval with few autophagosomes in negative control of AD-Null vehicle group (N). The autophagosomes and damaged mitochondria were significantly increased in AD-Null vehicle mice treated with MPTP group (NM). There are a large number of abnormal morphology mitochondria and some autophagosomes in AD-r-Txn1-shRNA-Null mice (S). The changed morphology mitochondria and autophagosomes were increased in AD-r-Txn1-shRNA-Null mice treated with MPTP (SM). Red arrow indicated the damaged mitochondria. Yellow arrow indicated autolysosomes with double membrane structures (**A**). Qualitative analysis of the number of autophagosome per 10 × 10 μm^2^ in the SNpc in MPTP-treated mice under TEM (**B** and **C**). The expressions of LC3 II/I and p62 in the SNpc in mice treated with MPTP were detected by Western blot analysis. The MPTP-increased expression of LC3 II/I and p62 was suppressed in Trx-1 overexpressing transgenic (Tg) mice (**D**, **E**) and was further increased in Trx-1 knockdown in the SNpc in mice (**F**, **G**). Each bar represents the mean ± SEM (n = 6). *P < 0.05, **P < 0.01, ***P < 0.001, statistically significant.

MPTP leads to neuronal oxidative stress and autophagic impairment. Mitochondrial dysfunction and impaired autophagy are features in both familial and sporadic PD [14]. However, the effect of Trx-1 on autophagy after MPTP treatment has not been reported. Mice of Trx-1 overexpression or Trx-1 knockdown in the SNpc were administrated with MPTP and the autophagosomes were examined by using electron microscopy (Fig. 4A). The autophagosomes (indicted by yellow arrow) contained mitochondria without crest structure and with a double membrane (indicted by red arrow) were increased, which were suppressed in Trx-1 overexpressing mice (Fig. 4B; two-way ANOVA: MPTP: F_(1,20)_ = 115.6, *P* < 0.001; genotype: F_(1,20)_ = 156.6, *P* < 0.001; interaction: F_(1,20)_ = 38.04, *P* < 0.001), and were further increased in Trx-1 knockdown mice (Fig. 4C; two-way ANOVA: MPTP: F_(1,20)_ = 40.5, *P* < 0.001; genotype: F_(1,20)_ = 53.39, *P* < 0.001; interaction: F_(1,20)_ = 1.389, *P* > 0.05). LC3 is a central protein in substrate selection and autophagosome formation during autophagy and used as a quantitative index of autophagy activation [15, 16]. The conversion of LC3 I to LC3 II is an essential step of autophagosome formation. Thus, LC3 II/I is used for measuring autophagy. p62 is a polyubiquitin chain binding protein involved in ubiquitin proteasome degradation and binds both ubiquitylated proteins and LC3 on the phagophore, thereby targets diverse substrates for autophagy. Thus, p62 accumulation serves as an indicator of autophagic impairment. We detected the expression of LC3 II/I and p62. The expression of LC3 II was increased by MPTP, which was suppressed in Trx-1 overexpressing mice (Fig. 4D; two-way ANOVA: MPTP: F_(1,20)_ = 15.54, *P* < 0.01；genotype: F_(1,20)_ = 9.392, *P* < 0.01; interaction: F_(1,20)_ = 49.37, *P* < 0.001), and was further increased in Trx-1 knockdown mice (Fig. 4E; two-way ANOVA: MPTP: F_(2,18)_ = 13.61, *P* < 0.01；genotype: F_(1,18)_ = 36.34, *P* < 0.001; interaction: F_(2,18)_ = 1.156, *P* > 0.05). The expression of p62 was increased by MPTP, which was suppressed in Trx-1 overexpressing mice (Fig. 4F; two-way ANOVA: MPTP: F_(1,20)_ = 21.15, *P* < 0.01；genotype: F_(1,20)_ = 4.589, *P* < 0.05; interaction: F_(1,20)_ = 5.043, *P* < 0.05), and was further increased in Trx-1 knockdown mice (Fig. 4G; two-way ANOVA: MPTP: F_(2,18)_ = 11.1, *P* < 0.01；genotype: F_(1,18)_ = 31.33, *P* < 0.001; interaction: F_(2,18)_ = 0.1102, *P* > 0.05). These results suggest that MPTP induces the autophagic impairment and Trx-1 may play a role in reducing autophagic impairment through regulating LC3 II/I expression and degradation of p62 in MPTP- treated mice. The above results suggest that Trx-1 regulates autophagy induced by MPTP.

### Effect of Trx-1 on the expression of PINK1 and Parkin induced by MPTP

**Fig. 5:**
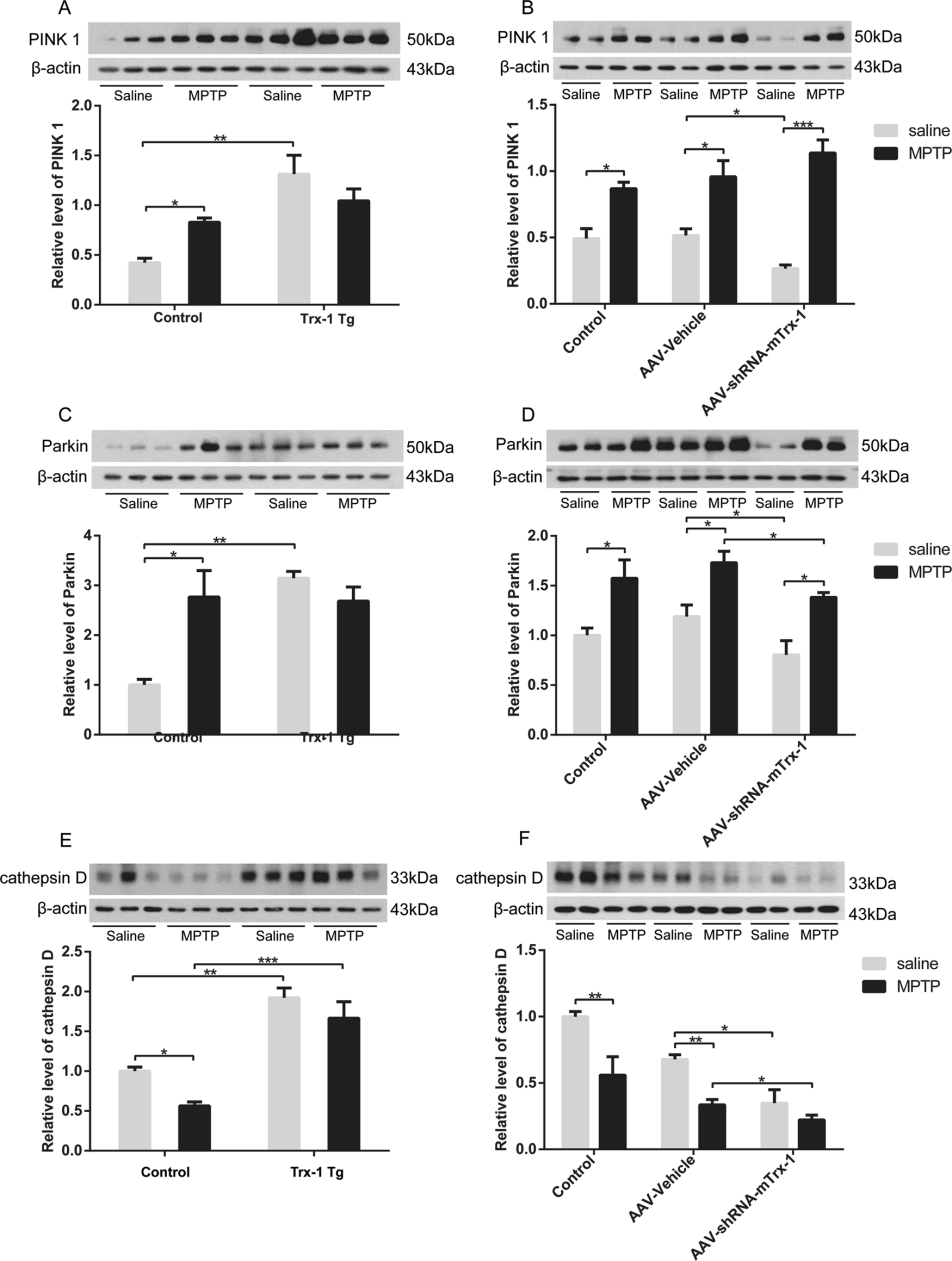
Effects of Trx-1 on expression of PINK1, Parkin and cathepsin D in the SNpc in MPTP-treated mice. The expression of PINK1 and Parkin in the SNpc were detected by Western blot analysis after administration with MPTP. The MPTP-increased PINK1 was reduced in Trx-1 overexpressing transgenic (Tg) mice **A** and was further increased in Trx-1 knockdown in the SNpc in mice **B**. The MPTP-increased Parkin was reduced in Trx-1 overexpressing transgenic (Tg) mice **C** and was further increased in Trx-1 knockdown in the SNpc in mice **D**. The expression of cathepsin D in the SNpc in mice treated with MPTP was detected by Western blot analysis. The MPTP-decreased cathepsin D was restored in Trx-1 overexpressing transgenic (Tg) mice **E** and was further decreased in Trx-1 knockdown in the SNpc in mice **F**. Each bar represents the mean ± SEM (n = 6). *P < 0.05, **P < 0.01, ***P < 0.001, statistically significant.

PINK1 and Parkin constitute a mitochondrial quality control function [17]. PINK1 is selectively stabilized on impaired mitochondria to activate Parkin, and Parkin is recruited from the cytosol to depolarized mitochondria to mediate the selective autophagic removal of the damaged organelle [18]. Thus, we explored the expression of PINK1 and Parkin in the SNpc after MPTP treatment. The expression of PINK1 and Parkin was increased by MPTP, which was suppressed in Trx-1 overexpressing mice (Figs. 5A and 5C; two-way ANOVA: A: MPTP: F_(1,20)_ = 22.45, *P* < 0.01；genotype: F_(1,20)_ = 0.3506, *P* > 0.05; interaction: F_(1,20)_ = 8.328, *P* < 0.05; C: MPTP: F_(2,18)_ = 0.2756, *P* > 0.05；genotype: F_(1,18)_ = 79.91, *P* < 0.001; interaction: F_(2,18)_ = 6.066, *P* < 0.05), and was further increased in mice of Trx-1 knockdown in the SNpc (Figs. 5B and 5D; two-way ANOVA: B: MPTP: F_(1,20)_ = 10.82, *P* < 0.05；genotype: F_(1,20)_ = 4.350, *P* > 0.05; interaction: F_(1,20)_ = 12.67, *P* < 0.05; D: MPTP: F_(2,18)_ = 4.561, *P* > 0.05；genotype: F_(1,18)_ = 32.36, *P* < 0.001; interaction: F_(2,18)_ = 0.01347, *P* > 0.05).The results suggest that Trx-1 is involved in expression of PINK1 and Parkin in MPTP-treated mice.

### Effect of Trx-1 on the expression of cathepsin D induced by MPTP

Lysosomes require a full range of proteases to carry out their function. Cathepsin D is ubiquitously expressed in almost all tissues with high levels and involved in PD [19]. Cathepsin B is a PD risk gene, it promotes fibrillar α-syn clearance, lysosomal function and glucocerebrosidase (GCase) activity in dopaminergic neurons [20]. Thus, we further detected the expression of cathepsin D and cathepsin B. As expected, expression of cathepsin D in the SNpc was significantly decreased after MPTP administration, which was restored in Trx-1 overexpressing mice (Fig. 5E; two-way ANOVA: MPTP: F_(1,20)_ = 63.98, *P* < 0.001；genotype: F_(1,20)_ = 7.519, *P* < 0.05; interaction: F_(1,20)_ = 0.5121, *P* > 0.05), and was further decreased in Trx-1 knockdown mice (Fig. 5F; two-way ANOVA: MPTP: F_(2,18)_ = 21, *P* < 0.001; genotype: F_(1,18)_ = 23.5, *P* < 0.001; interaction: F_(2,18)_ = 2.20, *P* < 0.5). Cathepsin B expression in the SNpc was significantly decreased after MPTP administration, which was restored in Trx-1 overexpressing mice (Fig. S2A; two-way ANOVA: MPTP: F_(1,20)_ = 45.4, *P* < 0.001; genotype: F_(1,20)_ = 4.62, *P* < 0.05; interaction: F_(1,20)_ = 4.08, *P* > 0.05), and was further decreased in Trx-1 knockdown mice (Fig. S2B; two-way ANOVA: MPTP: F_(2,18)_ = 96.88, *P* < 0.001; genotype: F_(1,18)_ = 207.2, *P* < 0.001; interaction: F_(2,18)_ = 20.18, *P* < 0.001). These data indicate that Trx-1 promotes lysosome function in MPTP-treated mice through restoring the expression of cathepsin D and cathepsin B. To further confirm lysosomes activity was regulated by MPTP, we also examined the expression of lysosomal-associated membrane protein 2 (LAMP2), a kind of membrane protein of lysosomes [21]. The expression of LAMP2 in the SNpc was significantly decreased after MPTP administration, which was restored in Trx-1 overexpressing mice (Fig. S3A; two-way ANOVA: MPTP: F_(1,20)_ = 53.19, *P* < 0.001; genotype: F_(1,20)_ = 4.936, *P* < 0.05; interaction: F_(1,20)_ = 8.468, *P* < 0.01), and was further decreased in Trx-1 knockdown mice (Fig. S3B; two-way ANOVA: MPTP: F_(2,18)_ = 51.62, *P* < 0.001; genotype: F_(1,18)_ = 231.3, *P* < 0.001; interaction: F_(2,18)_ = 20.91, *P* < 0.001).

### Effect of Trx-1 on mRFP-GFP-LC3

The tandem fluorescent mRFP-GFP-LC3 has been implicated in the maturation progression of autolysosomes from autophagosomes. The green puncta formation indicates the recruitment of LC3 to form the membrane of autophagic vesicles and will be quenched in a more acidic environment, such as autolysosomes, leaving the protein with only the mRFP fluorescent signal. Autophagosomes display both green and red fluorescence, thus LC3 puncta are yellow. The green, red and yellow fluorescence puncta were used to detect autophagy progression.

**Fig. 6:**
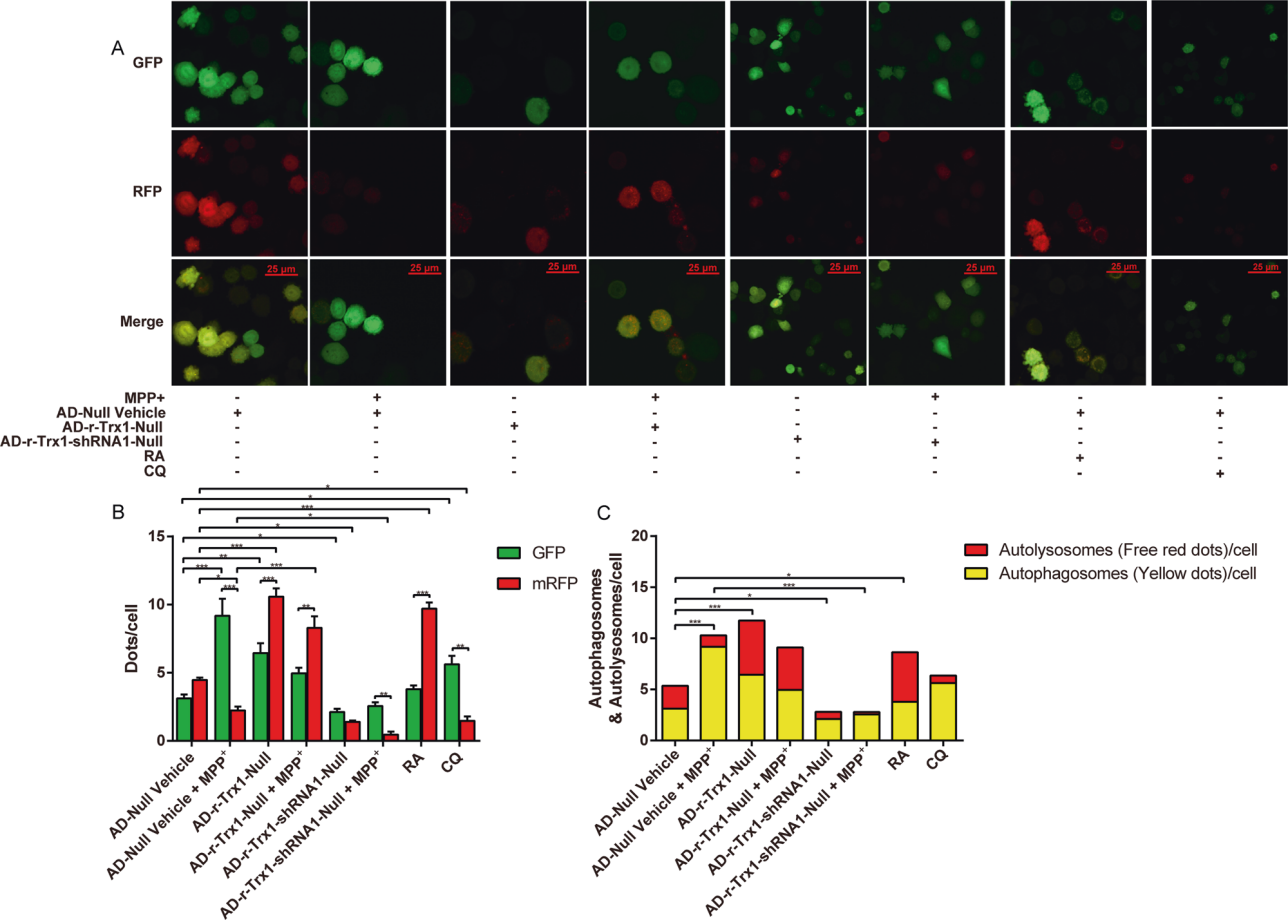
Effect of Trx-1 on the mRFP-GFP-LC3 in MPP^+^- treated PC12 cells. After transfection with AD-Null Vehicle, AD-r-Txn1-Null, AD-r-Txn1-shRNA1-Null for 24 h subsequently transfected with GFP-mRFP-LC3 plasmid at MOI of 1.26×10^10^ pfu/ml for 24 h and then treated with 0.3 mM MPP^+^ or 200 μM RA, 25 μM CQ for 24 h. GFP and RFP signals were visualized using confocal microscopy. The fraction of RFP/GFP signals was analyzed by the Mander’s coefficient using the ImageJ JACoP plugin. Magnification, 40×; bar, 25 μm (**A**). The GFP/RFP dots in Trx-1 overexpressing or knockdown PC12 cells treated with 0.3 mM MPP^+^ (**B**). The autolysosomes/autophagosomes dots in Trx-1overexpressing or knockdown PC12 cells treated with 0.3 mM MPP^+^ (**C**). Each bar represents the mean ± SEM (n = 3). *P < 0.05, **P < 0.01, ***P < 0.001, statistically significant.

To further monitor Trx-1 effect on the autophagy-lysosomes pathway, AD-Null Vehicle, AD-r-Trx1-Null, AD-r-Trx1-shRNA1-Null, and AD-GFP-mRFP-LC3 were transfected in PC12 cells. Compared with the Vehicle cells, the mRFP-GFP-LC3 showed a green fluorescent signal in cells treated with MPP^+^ and a red fluorescent signal in Trx-1 overexpressing cells as well as more green fluorescence signal in Trx-1 knockdown cells at the present and absent of MPP^+^, suggesting autophagosomes induced by MPP^+^ were regulated by Trx-1. Rapamycin (RA) and chloroquine (CQ) were used to indicate the autophagy promotion or inhibition (Fig. 6A). These results further suggest that Trx-1 regulates autophagy-lysosomes pathway in MPTP-treated mice (Fig. 6B; one-way ANOVA: F_(7,80)_ = 34.29, *P* < 0.001; Fig. 6C; one-way ANOVA: F_(7,80)_ = 19.69, *P* < 0.001).

## Discussion

α-syn is the major component of Lewy body, which is the histopathological hallmark of PD. Protein aggregates and damaged mitochondria represent key pathological markers of PD. Emerging studies have revealed that autophagy is implicated in the occurrence and development of PD [22]. Trx-1 has been reported to protect neurons in PD mice. In the present study, we found effects of Trx-1 on expression of α-syn and autophagy in PD.

At first, we found that MPTP administration induced motor deficits and decreased TH expression in the SNpc in mice, which were attenuated in Trx-1 overexpressing mice and were aggravated in Trx-1 knockdown mice (Figs. 1 and 2). Secondly, expression of α-syn protein and mRNA was increased by MPTP, which was reduced by Trx-1 overexpression, and increased further by Trx-1 knockdown (Fig. 3 and Fig. S1). Actually, the expression of α-syn was increased by MPTP in transcription and protein levels, which was coincident with previous study [23]. Anyway, Trx-1 could eliminate α-syn accumulation in MPTP-treated mice.

The electron microscope analysis showed that autophagosomes including impaired mitochondria were induced by MPTP in the SNpc, which were reduecd in Trx-1 overexpressing mice, and were enhanced in Trx-1 knockdown mice (Fig. 4). Reactive oxygen species (ROS) generated by MPP^+^ induces a strong autophagic response in dopaminergic cell lines, primary cortical and nigrostriatal neurons. The oxidative regulations of autophagy occur in all processes of autophagy, from induction, phagophore nucleation, phagophore expansion, autophagosome maturation, cargo delivery to the lysosome and degradation [24]. Indeed, ROS may have a negative impact on the autophagy machinery. Trx-1 is a critical antioxidant protein that regulates a wide range of cell processes [25]. Our previous study showed that Trx-1 recovered glutathione (GSH), and suppressed ROS in MPTP-treated mice [12, 13]. Thus, Trx-1 may play an important role in inhibiting ROS to improve autophagy induced by MPTP.

PINK1 and Parkin have been confirmed their roles in autophagy through their effects on protein aggregation, endolysosomal trafficking defects and mitochondria homeostasis respectively [26]. PINK1 recognizes and anchors to the outer membrane of impaired mitochondria, then recruits Parkin on the impaired mitochondria and promotes their elimination [27]. Mutated PINK1 and Parkin genes are involved in PD [28]. PINK1 and Parkin are accumulated in Lewy body together with α-syn [29]. Our results showed that the expression of PINK1 and Parkin was induced by MPTP, which was suppressed in Trx-1 overexpressing mice and was further increased in Trx-1 knockdown mice (Fig. 5A–D). These results indicate that Trx-1 is involved in regulating expression of Parkin and PINK1 in PD mice. LC3 II expression is markedly elevated in nigral PD samples [27, 30]. LC3 membranes are found to directly fuse with the outer mitochondrial membrane before incorporate into autophagosomes for degradation [31, 32]. These segregated mitochondria are selectively ubiquitinated via PINK1-mediated recruitment of Parkin on the mitochondrial surface, which are subsequently sequestered via p62 recruitment to lysosome for LC3-mediated autophagic degradation [33]. p62, a autophagy receptor, is defined as a new family of autophagy-related proteins that serves to target protein aggregation, mitochondria, intracellular pathogens and other cargoes to the core autophagy machinery via an LC3-interacting region (LIR)-motif [10]. When autophagy is inhibited, p62 accumulates because its degradation in autolysosomes is blocked [34]. Damaged mitochondria accumulated with PINK1 and Parkin are bound by the autophagic proteins p62 and LC3, resulting in degradation of mitochondria by mitophagy [7]. In the present study, baseline of LC3 II was increased, and the increase of LC3 II induced by MPTP was blocked in Trx-1 overexpressing mice (Fig. 4D). In contrast, baseline of LC3 II was decreased, and the increase of LC3 II induced by MPTP was further enhanced in Trx-1 knockdown mice when compared with lower baseline of LC3 II (Fig. 4E). p62 was increased by MPTP, which was inhibited in the Trx-1 overexpressing mice and was further increased in Trx-1 knockdown mice (Fig. 4F, G). These results further demonstrated that overexpression of Trx-1 promoted autophagy through regulating LC3 II and p62.

The autophagy-lysosome pathway plays a fundamental role in cellular physiology [35, 36]. This catabolic process is beneficial to eliminate harmful materials, such as misfolded proteins and damaged organelles, meanwhile provides nutrients to support cellular homeostasis and survival. Lysosomes are vital organelles in the endomembrane system of animal cells containing a diverse array of hydrolases, and the fusion of autophagosomes with lysosomes is one of the core steps in autolysosome maturation [37, 38]. Lysosomal dysfunction is recognized as one of the major pathological processes in neurodegenerative diseases, including Alzheimer disease (AD) and PD [39, 40]. Recent study reported that cathepsin D was decreased in plasma of patients with PD and was considered as a candidate for early-diagnosis plasma biomarker for PD [41]. Post-mortem studies showed the hindered autophagy-lysosomal process, increased LC3 II expression and decreased cathepsin D level [42]. In addition, the activities of lysosomal enzymes, such as GCase and the protease cathepsin D, were decreased in the SNpc and frontal cortex of patients with PD and patient with dementia of Lewy body [43]. In models of PD, lysosomal dysfunction resulted from cathepsin D haploinsufficiency promotes the cell-to-cell transmission of α-syn aggregates [44]. Moreover, cathepsin D-positive lysosomes are the only lysosomes performing a proteolytic activity in neurons [45]. Cathepsin D affects α-syn processing [46]. Boosting lysosomal cathepsin D activity not only enhanced α-syn clearance in neurons and tissues of human and murine, but also restored endo-lysosome and autophagy function [45]. The expression of PINK1, Parkin and cathepsin D is related with Trx-1 expression in diabetic diseases and AD [47–49]. Our results showed that decreased expression of cathepsin D by MPTP was restored in Trx-1 overexpressing mice and was further decreased in Trx-1 knockdown mice (Fig. 5E and F). In addition, we also detected expression of cathepsin B and LAMP2. Their changes were similar with cathepsin D (Figs. S1, S2). Importantly, under the microscope, we observed that mRFP-GFP-LC3 emitted a stronger red fluorescent signal in Trx-1 overexpressing PC12 cells treated with MPP^+^ or Trx-1 overexpressing PC12 cells, however, a stronger green fluorescent signal in Trx-1 knockdown in PC12 cells with or without MPP^+^ treatment (Fig. 6). These results showed that Trx-1 promoted autophagy-lysosome pathway via restoring lysosome function.

**Fig. 7:**
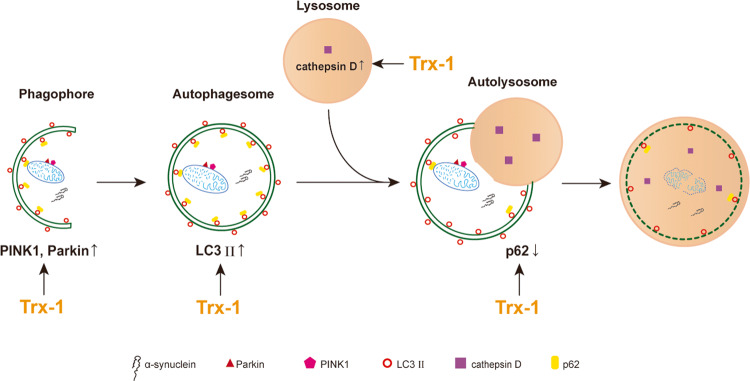
Trx-1 promotes the clearance of α-syn induced by MPTP through regulating autophagy-lysosomal pathway. Trx-1 inhibits increase of PINK1, Parkin and LC3 II/I, as well as restores expression of cathepsin D, finally decreases α-syn with p62 induced by MPTP.

Pathological α-syn not only disrupts normal synaptic function, but also interferes with the autophagy-lysosomal pathway and mitochondrial function [50, 51]. Our results showed that α-syn accumulation and impaired autophagy-lysosomal pathway induced by MPTP were rescued by Trx-1 overexpression. Thus, Trx-1 plays the roles in the clearance of α-syn in MPTP-treated mice through improving autophagy-lysosomal pathway (Fig. 7). Trx-1 may act as the potential target of autophagy-lysosomal pathway for the curative treatment of PD.

## Materials and methods

### Animals

Eight-week-old (18–22 g) male C57BL/6 wild-type (WT) mice (Chongqing, China) and C57BL/6 human Trx-1 overexpression transgenic (Trx-1 Tg) mice (constructed by Cyagen Biosciences Inc., Guangzhou, China) were housed (5 mice/cage) in an air-conditioned room (24 ± 1 °C) with a 12 h light/dark cycle and free access to food and water.

Mice were anesthetized with intraperitoneal injection of pentobarbital sodium salt (50 mg/Kg) before surgery and each mouse received two unilateral SNpc injections (AP - 3.08 mm, DV - 4.6 mm and Lat ± 0.75 mm, AP relative to bregma, DV relative to skull surface and Lat relative to midline, injection rate 0.1 μL/min) of the 0.6 μL recombinant r-AAV9-ZsGreen-mTrx-1-shRNA (AAV-shRNA-mTrx-1) or r-AAV9-ZsGreen-vehicle (AAV9 vehicle) (Guangzhou ribobio Co. Ltd., Guangzhou, China).

All protocols and procedures were approved by the animal ethics council of Kunming University of Science and Technology, and are in accordance with the National Institutes of Health Guide for the Care and Use of Laboratory Animals. The license was approved by the local Committee on Animal Use and Protection of Yunnan province (No. LA2008305).

### Drugs and treatment

For the Trx-1 overexpressing mice experiment, mice were randomly divided into four groups (n = 15 per group), control group, MPTP group, Trx-1 overexpressing group (Trx-1 Tg) and Trx-1 Tg + MPTP group. Mice in the control and Trx-1 overexpressing groups were administered saline daily over a period of 7 days. Mice in the MPTP and Trx-1 overexpressing + MPTP groups were given intraperitoneal injection of MPTP-HCl (dissolved in saline) at 30 mg/kg/d for 7 days, and the dose of MPTP-HCl was chosen in accordance with our previous study [13]. For the Trx-1 knockdown mice experiment, mice were divided into 6 groups (n = 15 per group), control group (C group), MPTP group (M group), AAV vehicle group, AAV vehicle + MPTP group, AAV-shRNA-mTrx-1 group, AAV-shRNA-mTrx-1 + MPTP group. After two weeks of AAV-shRNA-mTrx-1 injection with brain stereotactic apparatus, mice in the MPTP, AAV vehicle + MPTP, and AAV-shRNA-mTrx-1 + MPTP groups were given intraperitoneal injections of MPTP-HCl for 7 days. Mice in the control, AAV9 vehicle and AAV9-shRNA-mTrx-1 groups were administered saline daily for 7 days.

### Behavioral test

#### Rotarod experiment

A rotarod experiment requires mice to keep balance and move continuously on the rotating rod, which is widely used for assessment of limbs coordination ability. Before the formal experiment, the mice were trained once a day for three consecutive days. On the first day, mice were placed on the rotating rod at 5 rpm for 10 min. On the second day, mice were placed on the rotating rod at 12 rpm for 10 min. On the third day, mice were placed on the rotating rod at 18 rpm for 10 min. In the formal experiment, five mice were placed on the rotating rod to adapt for 5 s. After the mice were stabilized, the instrument was started to increase the rotating speed from 5 rpm to 40 rpm within 300 s. The falling time of each mouse was recorded, and the average value of the three times was taken as the average score in the rotarod experiment.

### Traction test

Mice were allowed to hold onto a horizontal wire, by their forepaws, and observed for 10 s. The hind limb placements were scored from 1 to 3, with the lowest score indicating the most severe deficit. Animals were assigned a score of 3 for gripping the wire with both hind paws, 2 for gripping the wire with one hind paw, and 1 for gripping the wire with no hind paw.

### Reagents

Antibodies against the following proteins were used in this study: TH (abcam, ab137869; 1:5000), Trx-1 (Proteintech, 14999-1-AP; 1:2000), α-syn (abcam, ab212184; 1:2000), PINK1 (Santa Cruz Biotechnology, sc-517353; 1:500), Parkin (Santa Cruz Biotechnology, sc-32282; 1;500), LC3I/II (Cell Signaling Technology, #4108; 1:1000), p62 (Proteintech, 66184-1-Ig; 1:2000), cathepsin B (Proteintech, 12216-1-AP; 1:2000) cathepsin D (Santa Cruz Biotechnology, sc-377299; 1:500), LAMP2 (Proteintech, 66301-1-Ig; 1:2000), β-actin (Proteintech, 81115-1-RR; 1;10000), Anti-Mouse IgG (H + L) Antibody, (SeraCare, 5450-0011; 1:10000), Anti-Rabbit IgG (H + L) Antibody (SeraCare, 5450-0010; 1:10000). Fluorophore-conjugated secondary antibodies were as follows: Goat anti-Rabbit IgG (H + L) Highly Cross-Adsorbed Secondary Antibody, Alexa Fluor™ 568 (Thermo Fisher, A-11036; 1:1000). Other reagents included MPTP-HCl (Sigma-Aldrich-Corporation, 23007-85-4), MPP^+^ (Sigma-Aldrich-Corporation, 36913-39-0), Rapamycin (MedChemExpress, HY-10219), Chloroquine phosphate (MedChemExpress, HY-17589), Antifading Mounting Medium with DAPI, (Solarbio, S2110).

### Immunofluorescence microscopy

The SNpc tissue (freezing microtome, 20 µm) were detected with anti-TH antibody (Abcam, ab137869) and anti-α-syn antibody (Abcam, ab212184) at a dilution of 1:200. Coronal sections (20 μm) of SNpc tissues were washed with PBS 3 times for 15 min and then were immersed into citrate antigen retrieval solution for 40 min (95°C water bath). Incubated tissue sections for 1.5 h in a blocking solution (0.3% TritonX-100, 10% goat serum in 0.01 M PBS) and then immunostained with a mixture of primary antibodies in a blocking solution at 4 °C on a shaker overnight. After washing in PBS 3 times, sections were incubated with corresponding fluorescent secondary antibodies (1:1000) for 1.5 h at room temperature in the dark and then washed with PBS 3 times. Sealed sections by antifading mounting medium with DAPI, observed and obtained images by fluorescence microscopy (JEOL1200CX).

### Electron microscopy

Some SNpc tissues were used for scanning electron microscopy. Thin (1 µm) sections were fixed in 2.5% glutaraldehyde, post-fixed in 1% osmium tetroxide and stained with 1% uranyl acetate from matching areas of experimental and visualized using an electron microscope (JEOL1200CX) at 100 kV.

### Cell culture and evaluation of fluorescent LC3 puncta

PC12 cells were cultured as described [13] and were treated with 200 μM Rapamycin (RA), 25 μM Chloroquine phosphate (CQ). The transfection with AD-Null Vehicle, AD-r-Trx1-Null, AD-r-Trx1-shRNA1-Null, and AD-GFP-mRFP-LC3 (Shanghai, Hanbio Biotechnology Co., Ltd, China) were using established protocols. The autophagy lysosomes pathway regulated by MPP^+^ in PC12 cells was imaged by using the laser scanning confocal microscope (LSM-700, Germany). The number of GFP and mRFP dots was determined by manual counting of fluorescent puncta in five fields from three different myocyte preparations with a 60 objective. The number of dots per cell was obtained by dividing the total number of dots by the number of nuclei in each microscopic field.

### Western blot analysis

The SNpc tissues were extracted using protein lysates, and the protein concentration was determined with the Bio-Rad protein assay reagent (Hercules, CA, USA). Protein samples were separated by 10–15% SDS-PAGE and transferred to a 0.45 μm olyvinylidene difluoride membrane (PVDF, Millipore Corp., Billerica, MA, USA). The membrane was washed in TBS-Tween 3 times for 15 min and blocked after transferring 2 h at room temperature in 10% skim milk (in phosphate buffered saline, pH 7.2, containing 0.1% Tween 20).

### Quantitative real-time polymerase chain reaction (RT-qPCR)

Total RNA was extracted from SNpc tissues from different groups using the RNAiso Plus reagent (TaKaRa, Japan, Cat. No. 108-95-2) according to the manufacturer’s instructions. cDNA was synthesized using the Revert Aid First stand cDNA Synthesis kit (ThermoFisher Scientific, UAB, K1622). To quantify the mRNA level of α-syn, RT-qPCR was performed on a CFX96 Touch thermocycler (Applied Biosystems) using SYBR Premix Ex Taq II (Applied Biosystems/ThermoFisher Scientific, UAB, A25742). Primer sequences for α-syn and GAPDH (Sangon Biotech, Shanghai, China) were used as follows: α-syn (Forward Primer): 5′-AGGGAGTCGTTCATGGAGTG-3′, α-syn (Reverse Primer): 5′-TACCCTTCTTCACCCTTGCC-3′; GAPDH (Forward Primer): 5′-AGGTCGGTGTGAACGGATTTG-3′, GAPDH (Reverse Primer): 5′-GGGGTCGTTGATGGCAACA-3′. PCR amplification was carried out at 95 °C for 30 s, followed by 45 cycles of 95 °C for 5 s and 55 °C for 30 s. GAPDH was used as an endogenous control to normalize differences. All fluorescence data were processed by a PCR post-data analysis software program. The differences of gene expression were analyzed with the 2 –ΔΔCT method.

### Statistical analysis

All results were expressed as means ± SEM. Statistical analysis was performed by using SPSS software. Western blot analysis results were analyzed by analysis of variance (ANOVA) by a post hoc Bonferroni multiple comparison test was used to compare different groups. *P* value less than 0.05 was considered as statistically significant difference. All blots are representative of experiments that were performed at least three times.

### Supplementary information

Supplementary materials

## Data Availability

Data and materials supporting the findings of this study are available within the paper.
