# Supplementary material for: Thioredoxin-1 decreases alpha-synuclein induced by MPTP through promoting autophagy-lysosome pathway
Source: Cell Death Discov. 2024 Feb 22;10:93. doi: 10.1038/s41420-024-01848-0 (PMC10884002; doi:10.1038/s41420-024-01848-0)

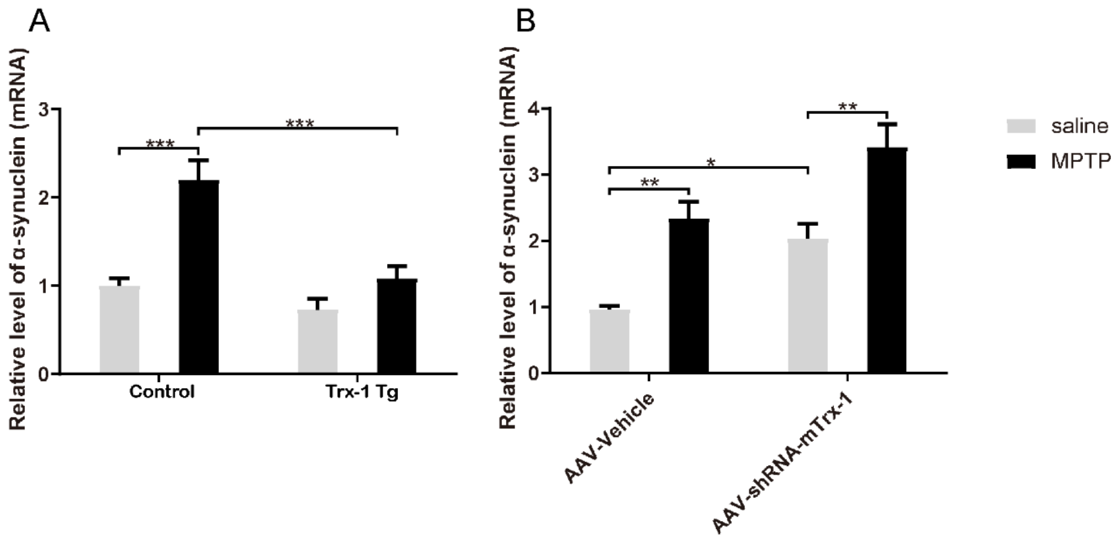

Supplementary Figure 1. Effect of Trx-1 on α-syn mRNA level in the SNpc in MPTP-treated mice.

The mRNA level of α-syn was increased MPTP, which was suppressed by Trx-1overexpressing **(A)**, and further increased by Trx-1 knockdown **(B)**. Each bar represents the mean ± SEM (n = 6). * $P < 0.05$, ** $P < 0.01$, *** $P < 0.001$, statistically significant.

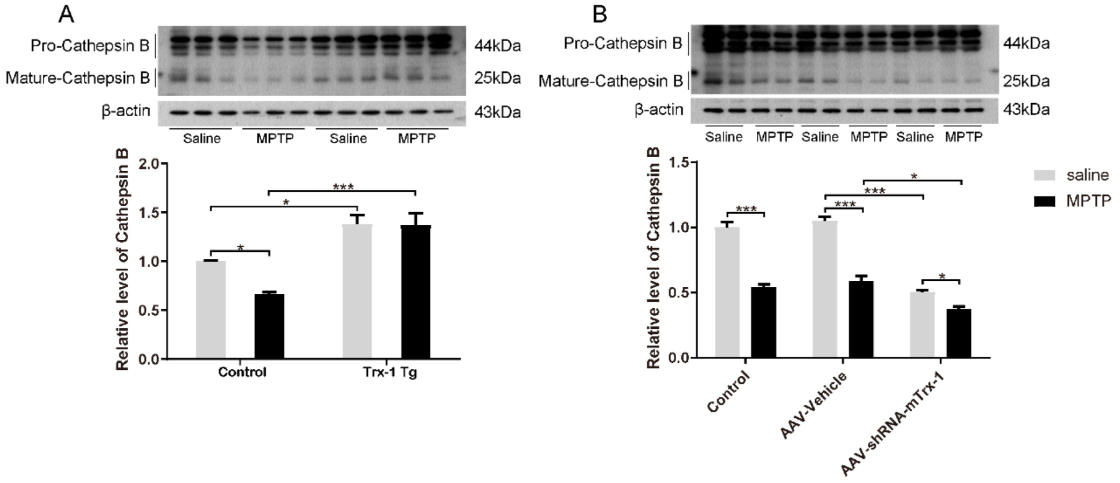

Supplementary Figure 2. Effect of Trx-1 on expression of cathepsin B in the SNpc in MPTP-treated mice.

The expression of cathepsin B in the SNpc was detected by Western blot analysis after administration with MPTP. The expression of cathepsin B was decreased by MPTP, which was restored in Trx-1 overexpressing transgenic (Tg) mice **(A)** and was further decreased in Trx-1 knockdown in the SNpc in mice **(B)**. Each bar represents the mean ± SEM (n = 6). * $P < 0.05$, *** $P < 0.001$, statistically significant.

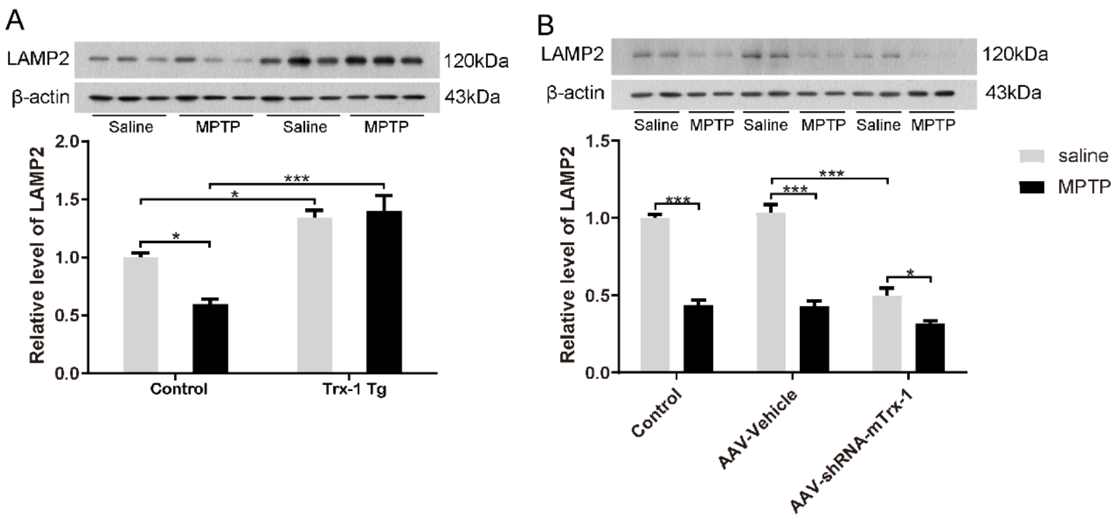

Supplementary Figure 3. Effects of Trx-1 on expression of LAMP2 in the SNpc in MPTP-treated mice.

The expression of LAMP2 in the SNpc was detected by Western blot analysis. The The expression of LAMP2 was decreased by MPTP, which was restored in Trx-1 overexpressing transgenic (Tg) mice **(A)** and was further decreased in Trx-1 knockdown in the SNpc in mice **(B)**. Each bar represents the mean ± SEM (n = 6). * *P* < 0.05, *** *P* < 0.001, statistically significant.

α-syn

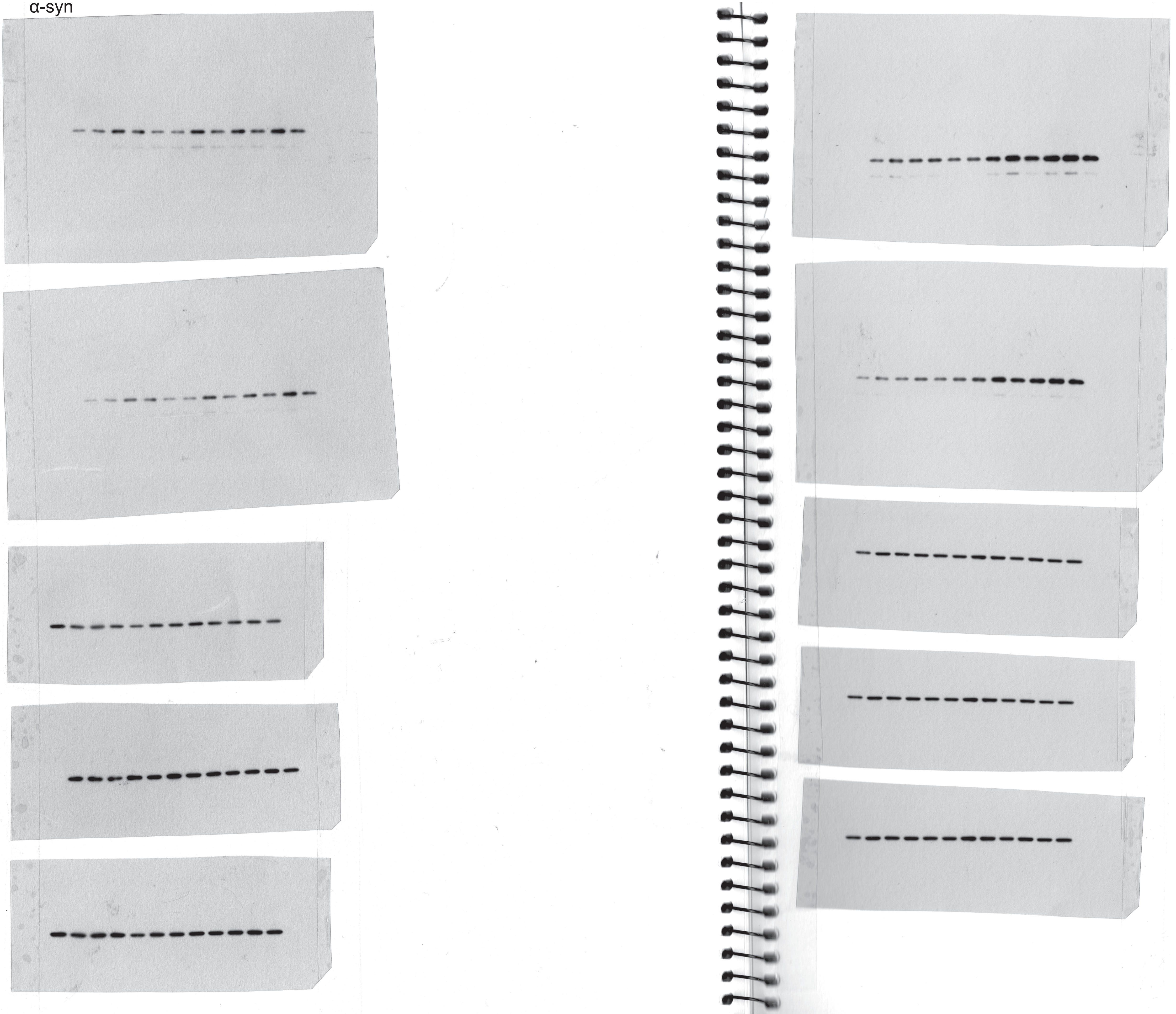

[illegible]

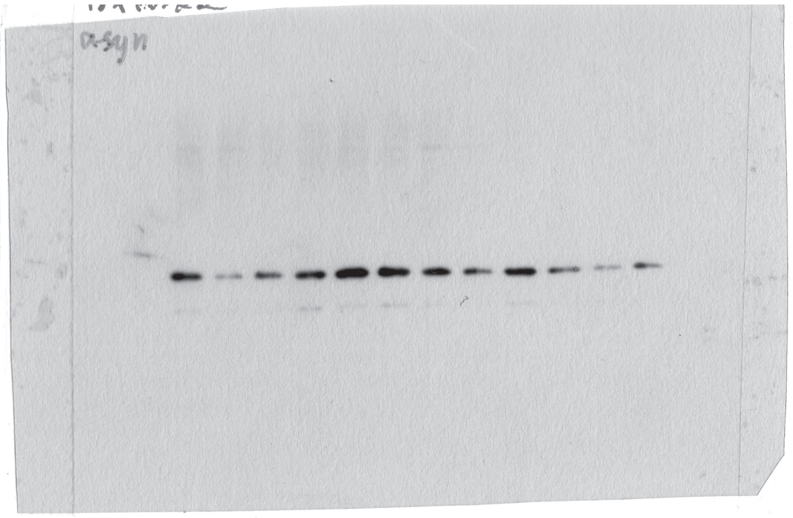
α-syn

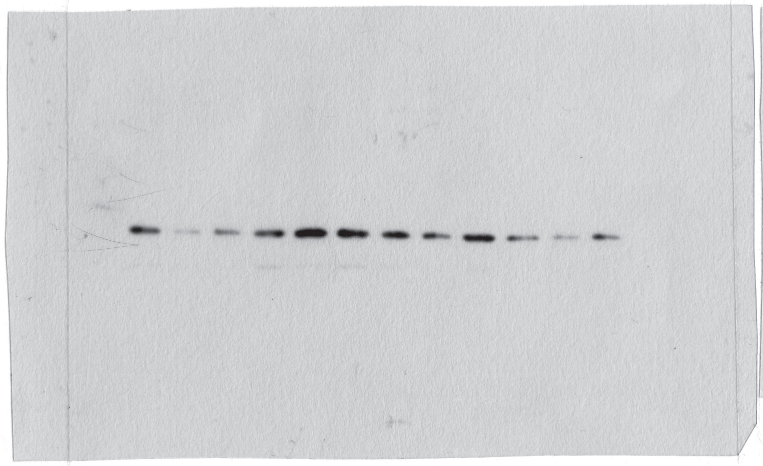

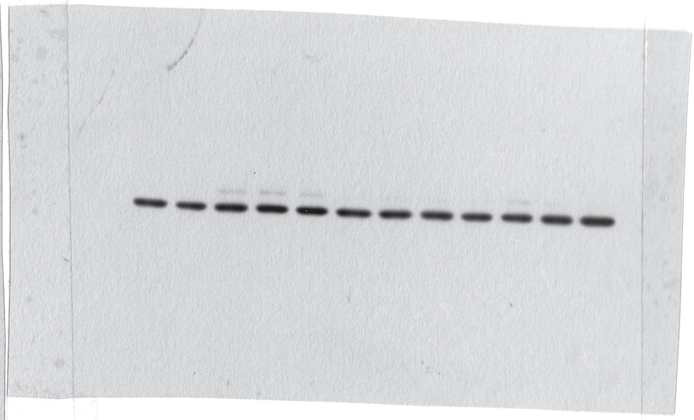

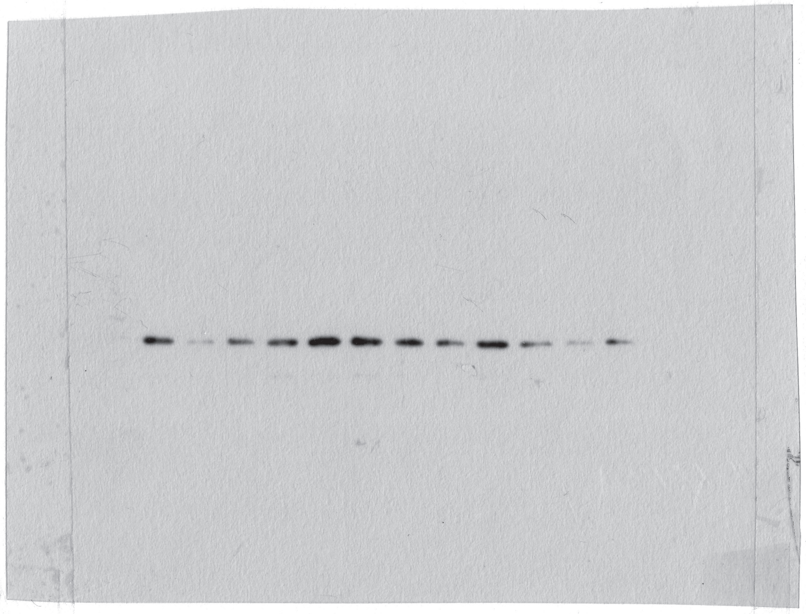

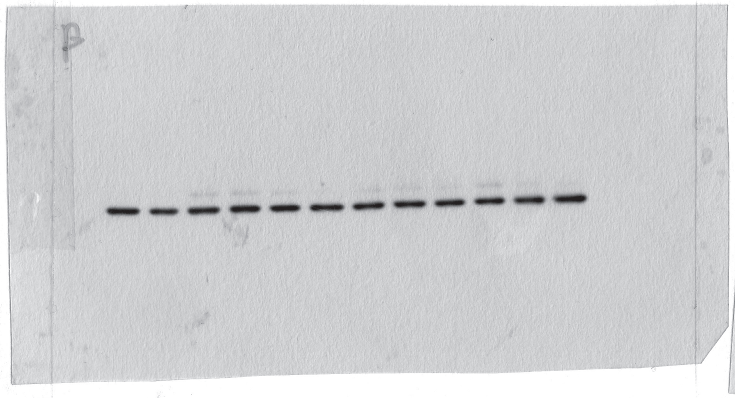
β

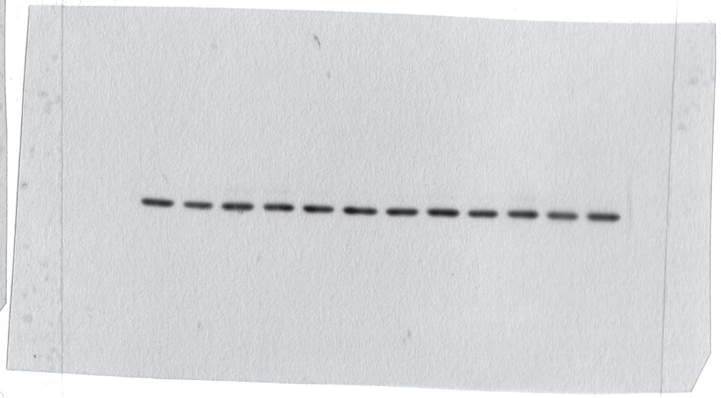

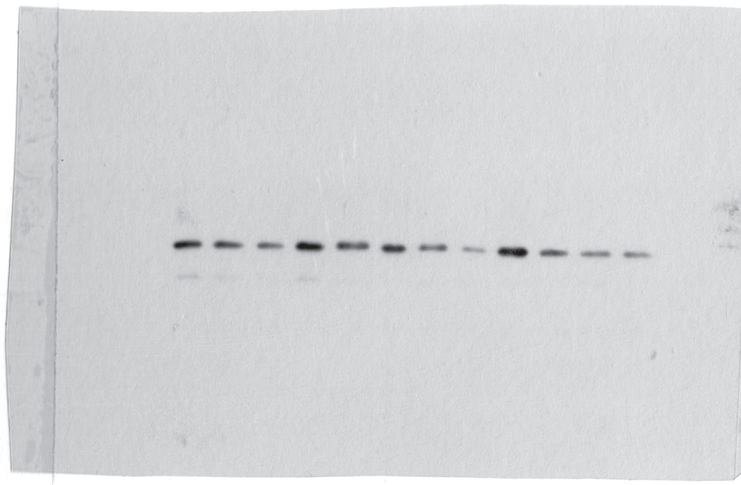

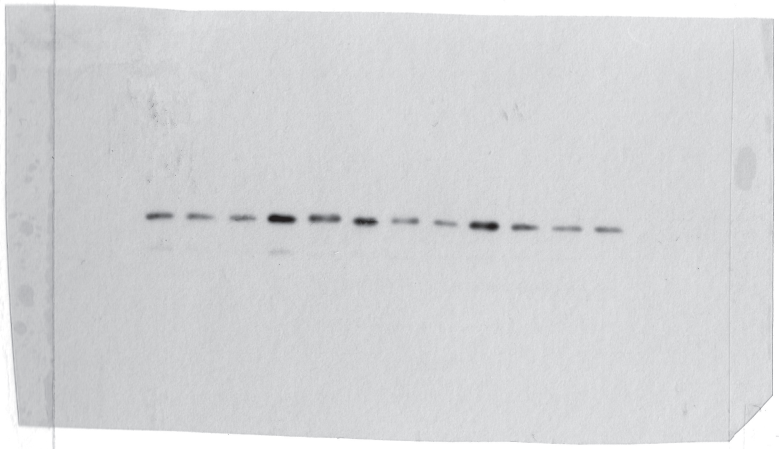

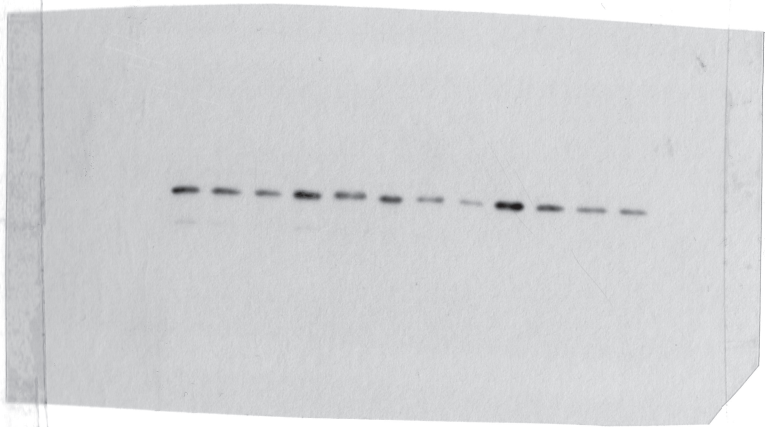

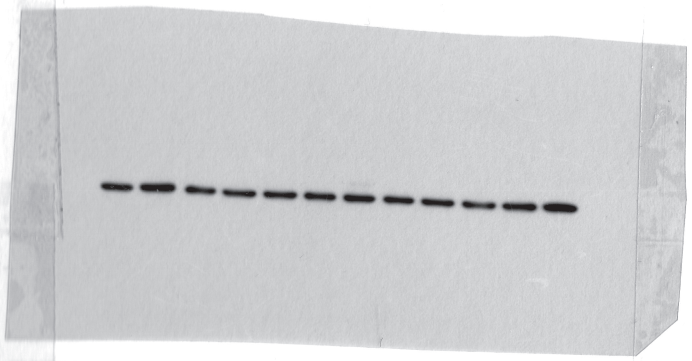

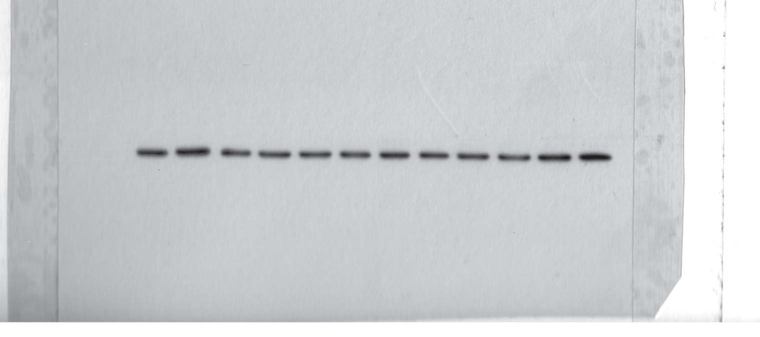

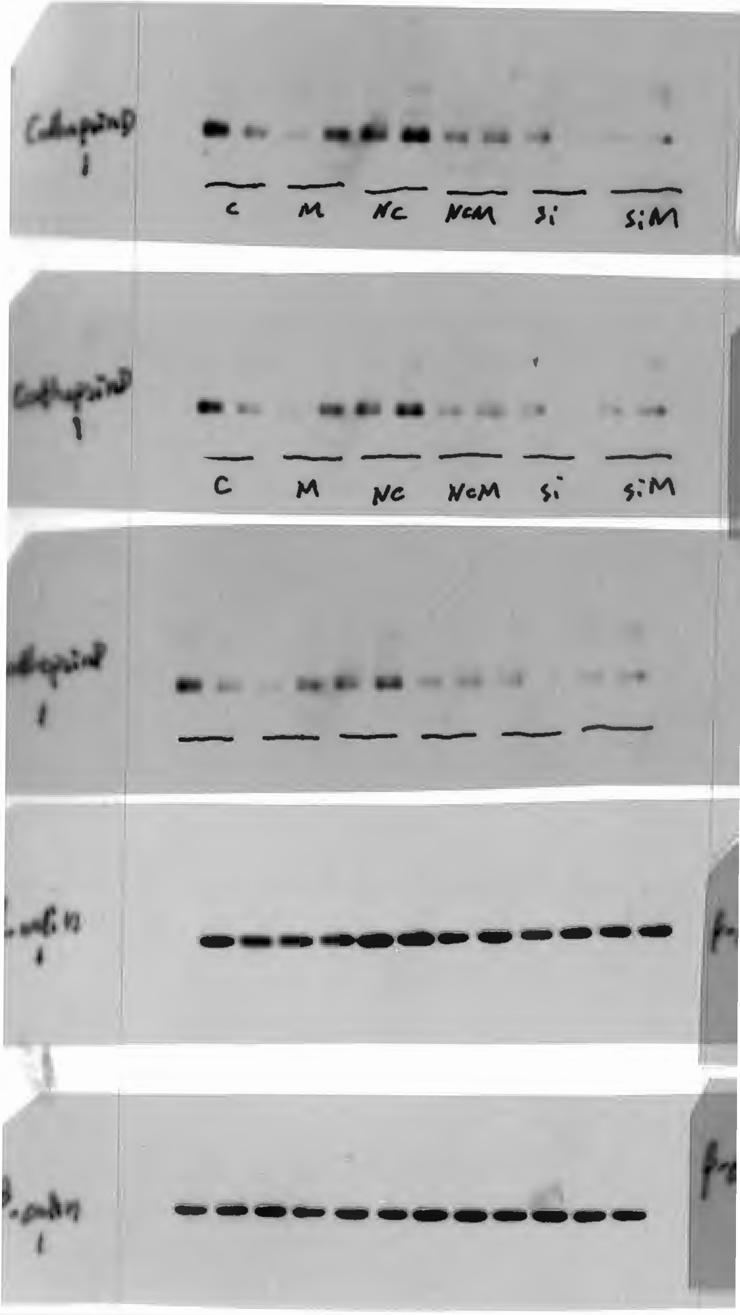
C
M
NC
NCM
si
siM
C
M
NC
NCM
si
siM

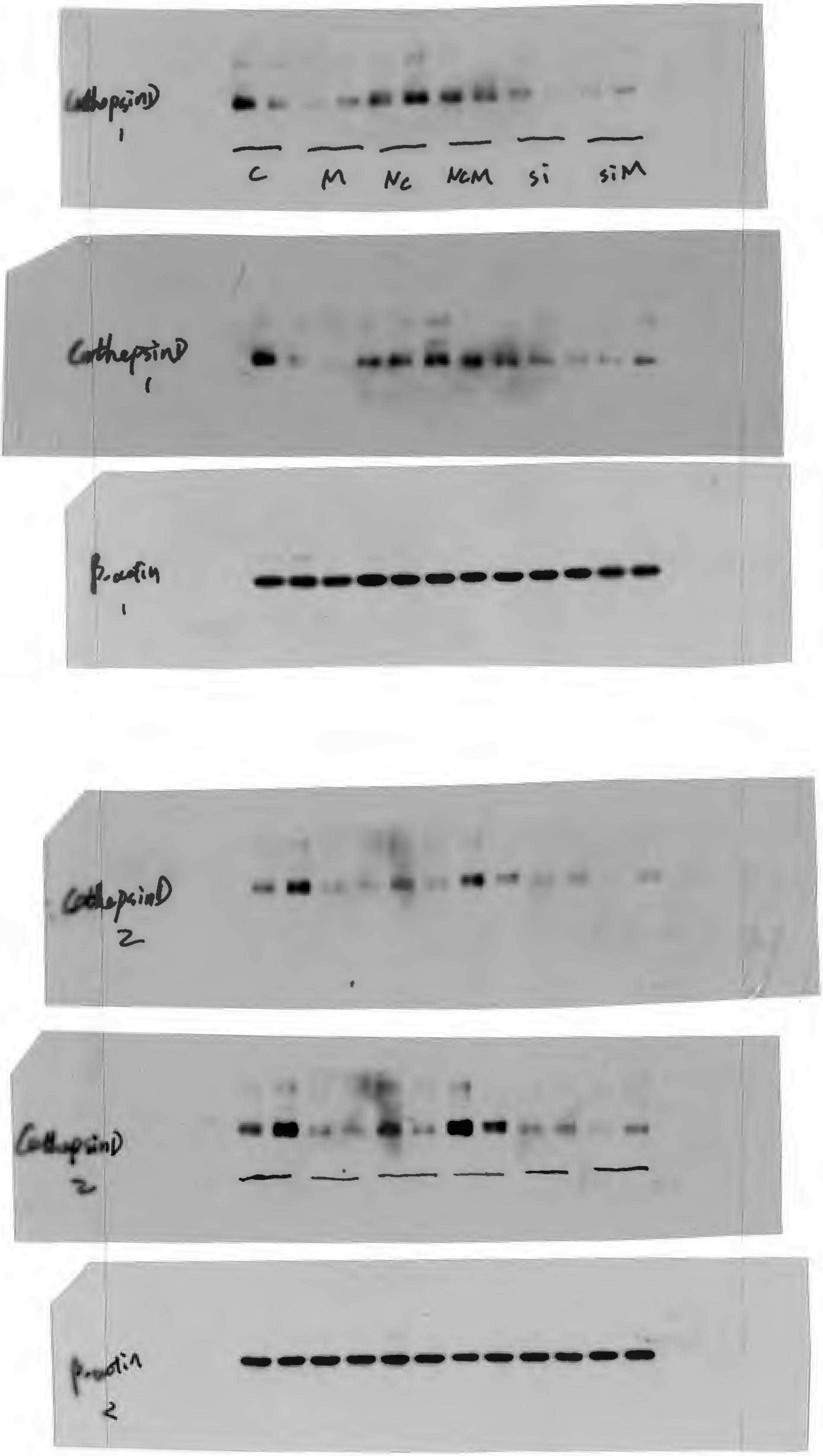
Cathepsin D
1
C M Nc NcM si siM
Cathepsin D
1
β-actin
1
Cathepsin D
2
Cathepsin D
2
β-actin
2

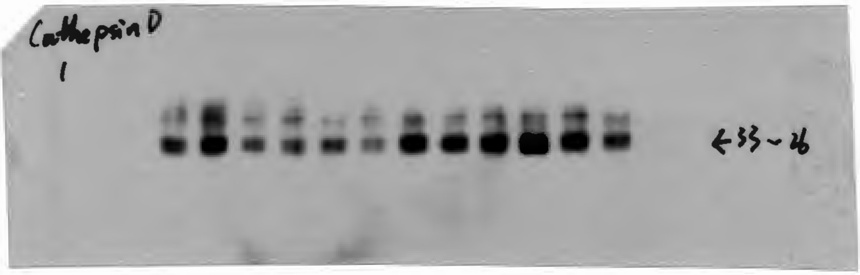
Cathepsin D
← 33~26
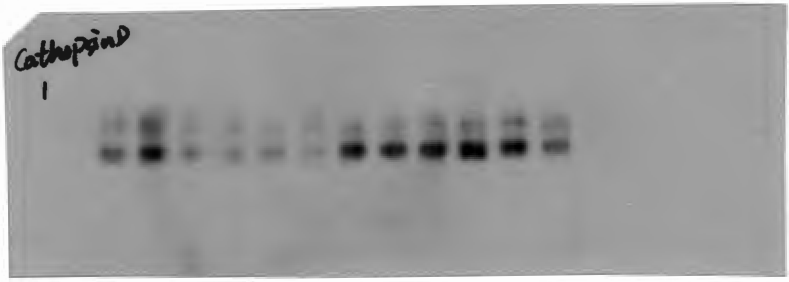
Cathepsin D
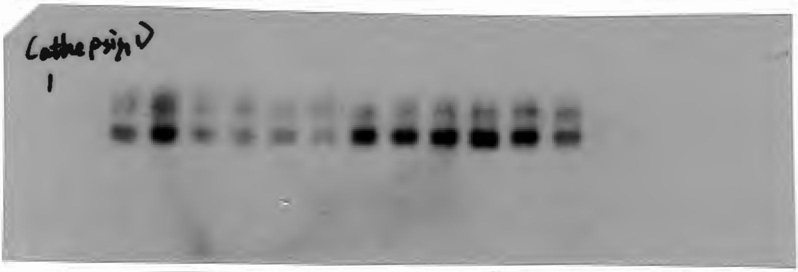
Cathepsin D
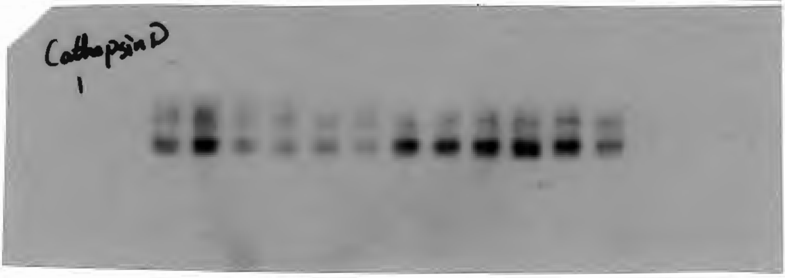
Cathepsin D
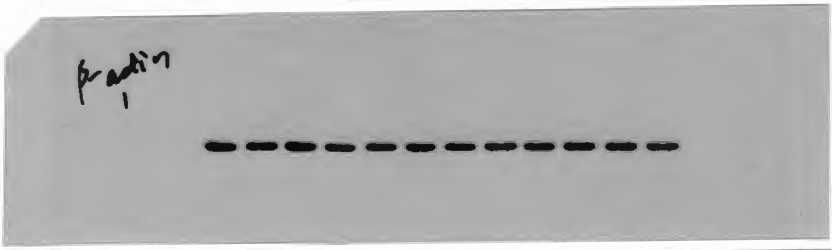
β-actin
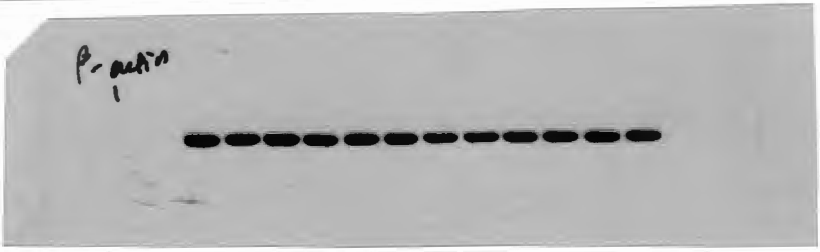
β-actin

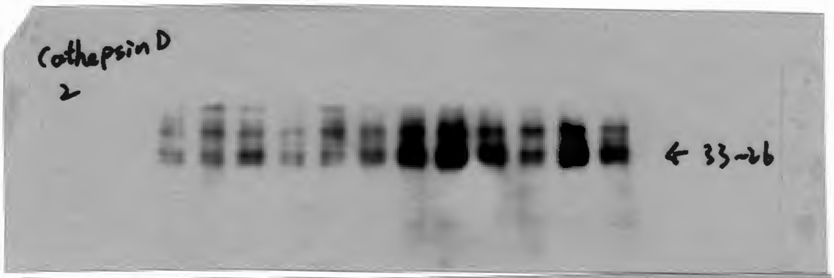
cathepsin D
2
← 33-26

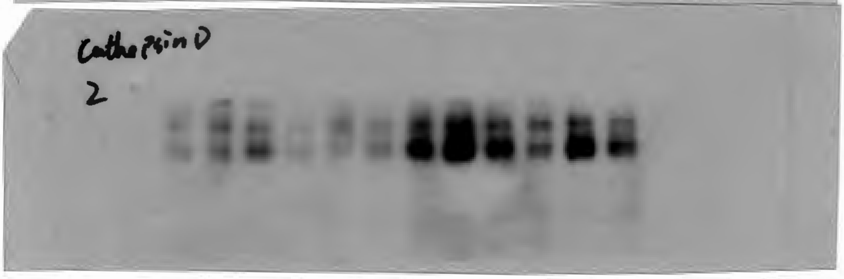
cathepsin D
2

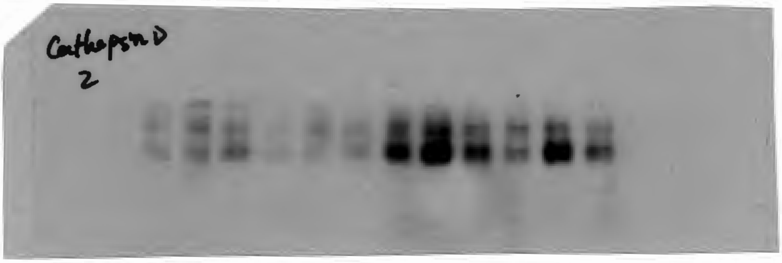
Cathepsin D
2

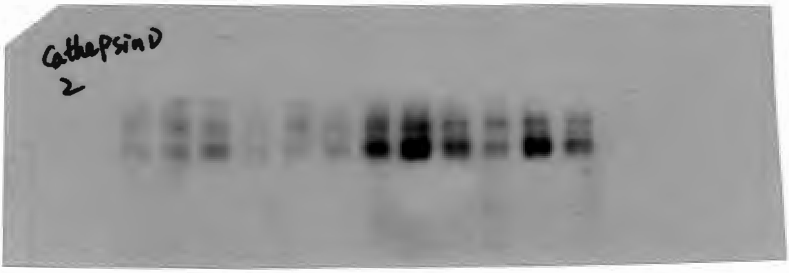
cathepsin D
2

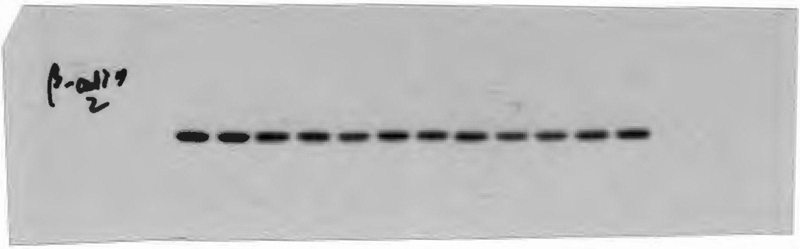
β-actin
2

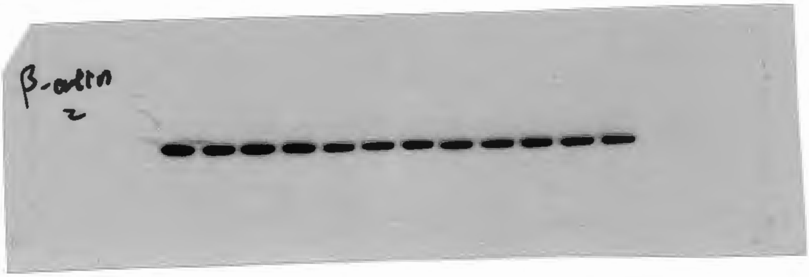
β-actin
2

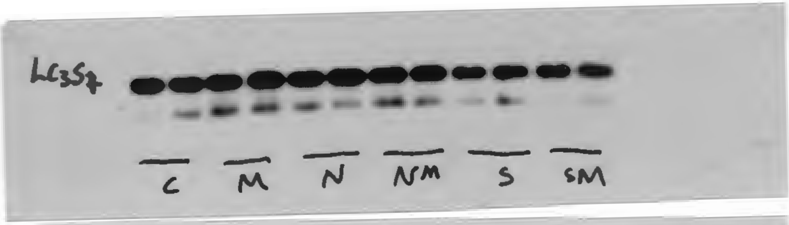
C
M
N
NM
S
SM

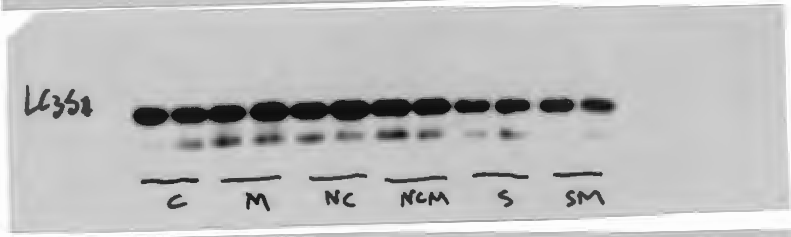
C
M
NC
NCM
S
SM

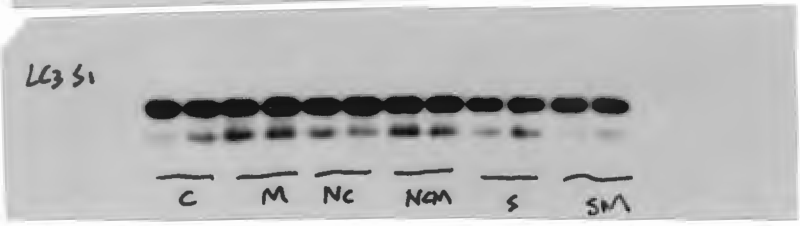
LC3 S1
C
M
NC
NCM
S
SM

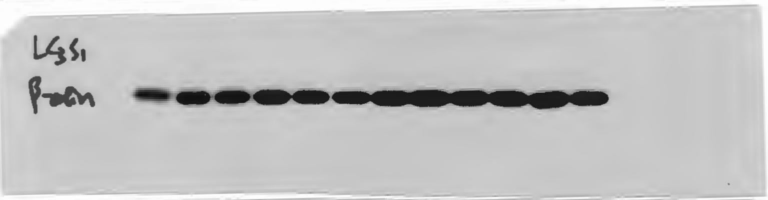
LC3S1
β-actin

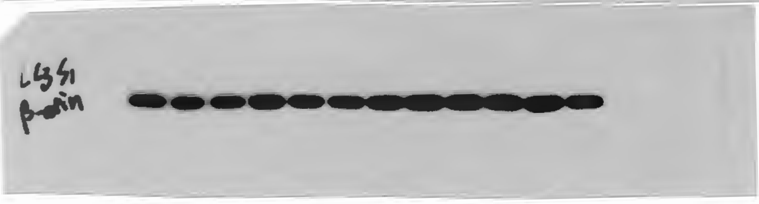
LC3S1
β-actin

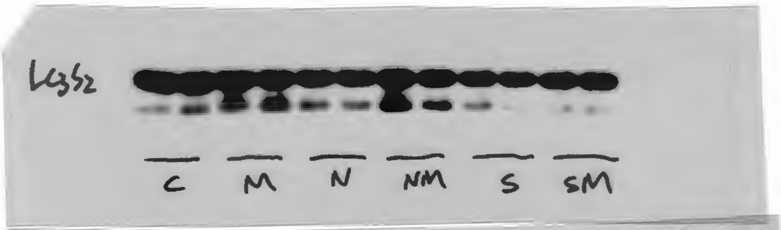
LC3S2
C
M
N
NM
S
SM

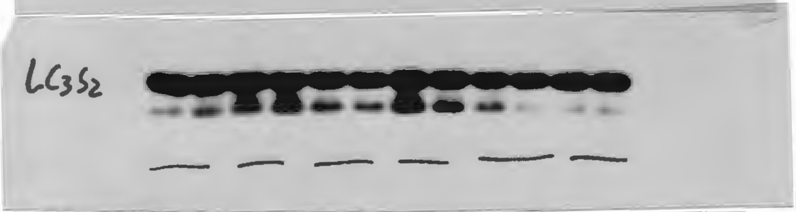
LC3S2

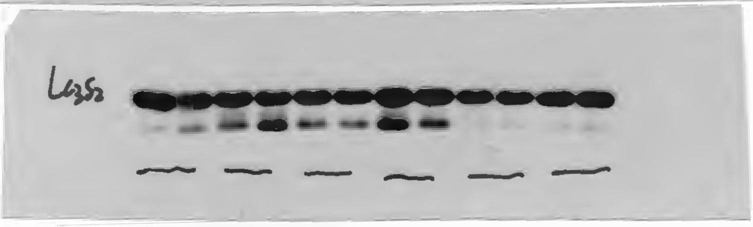
LC3S2

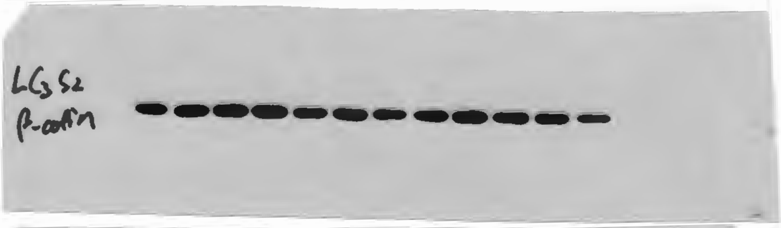
LC3S2
β-actin

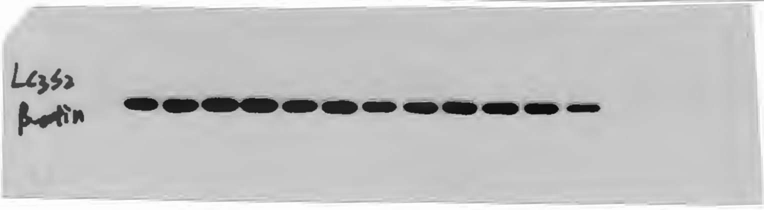
LC3S2
β-actin

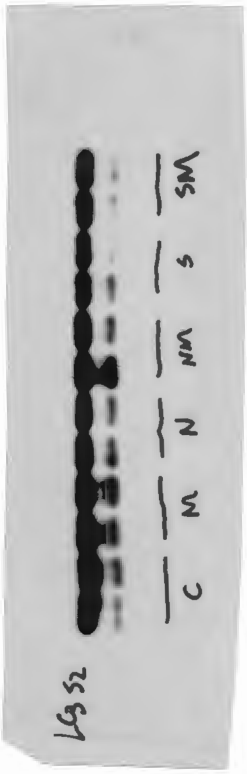
LC3S2
C
M
N
NM
S
SM

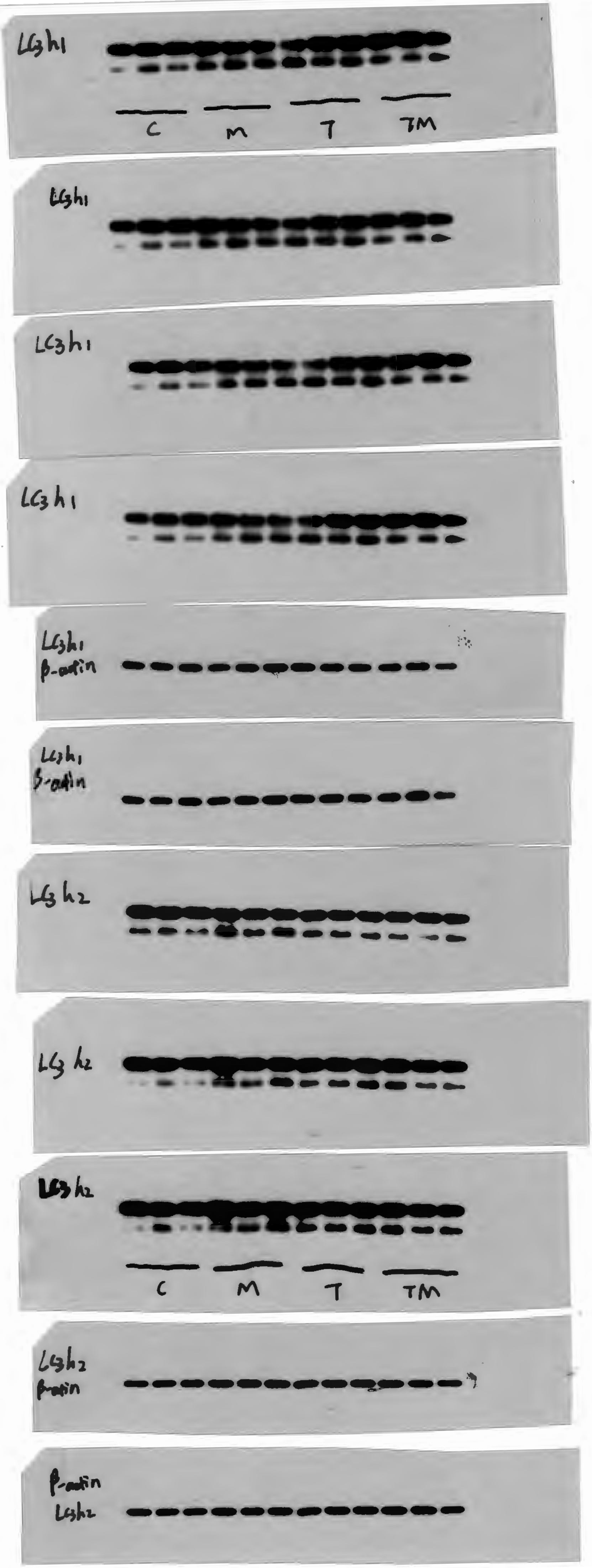
LC3h1
C
M
T
TM
LC3h1
LC3h1
LC3h1
LC3h1
β-actin
LC3h1
β-actin
LC3h2
LC3h2
LC3h2
C
M
T
TM
LC3h2
β-actin
β-actin
LC3h2

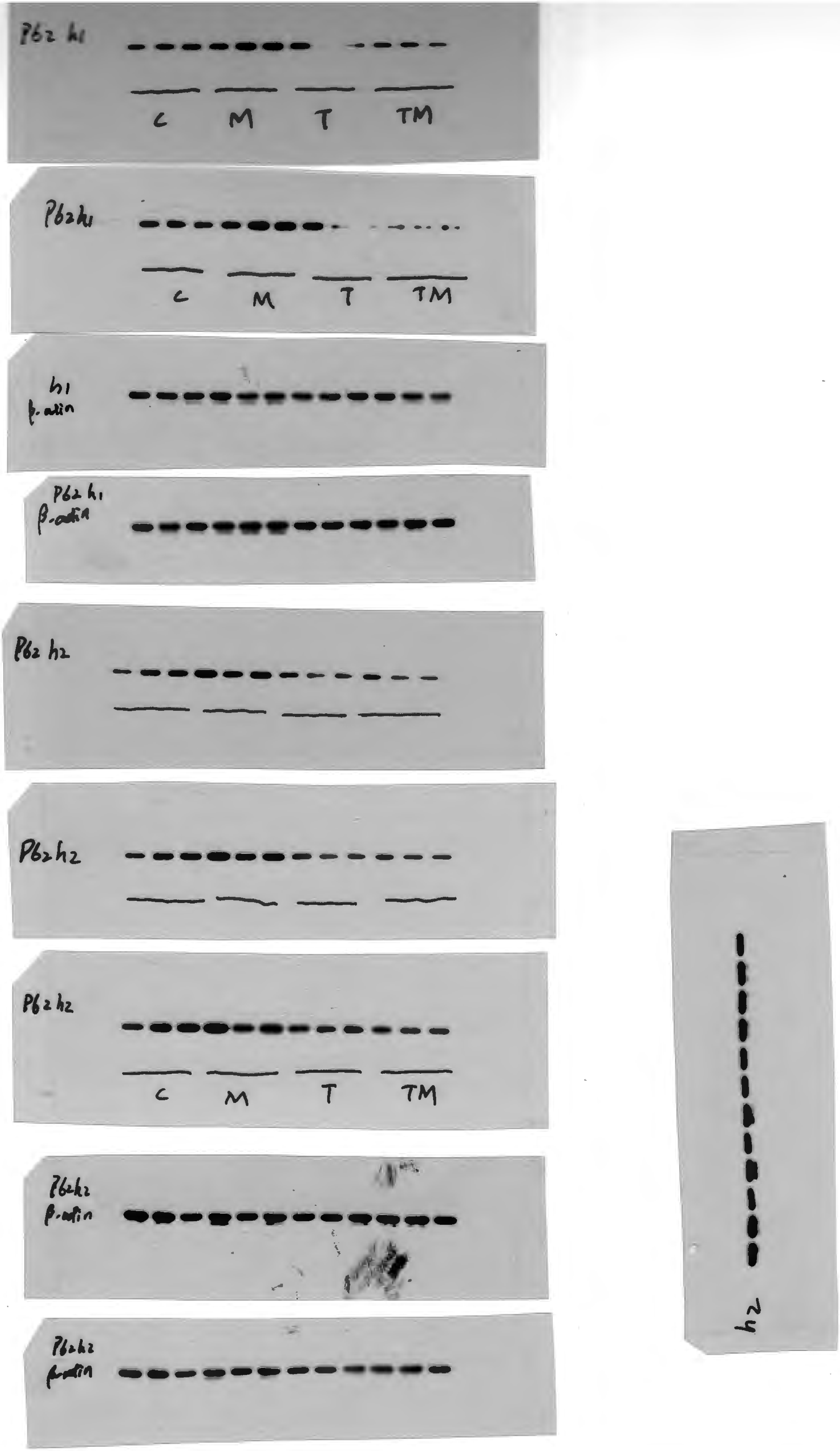

P62 h1
C M T TM
P62h1
C M T TM
h1 β-actin
P62 h1 β-actin
P62 h2
P62h2
P62h2
C M T TM
P62h2 β-actin
P62h2 β-actin
h2

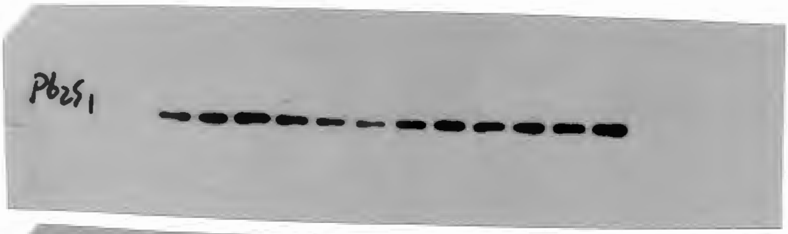
Pb2S1

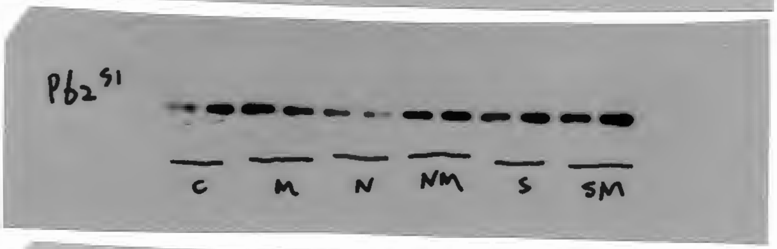
Pb2S1
C M N NM S SM

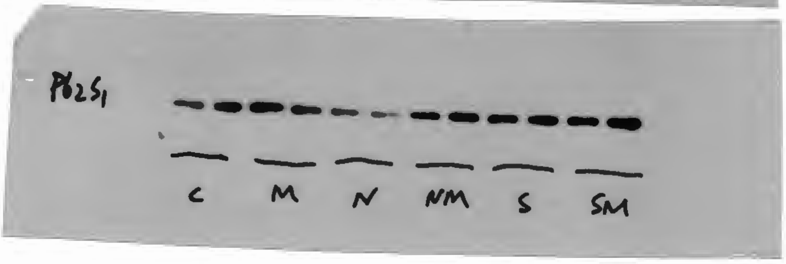
Pb2S1
C M N NM S SM

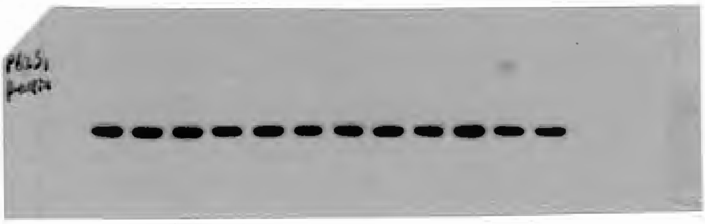

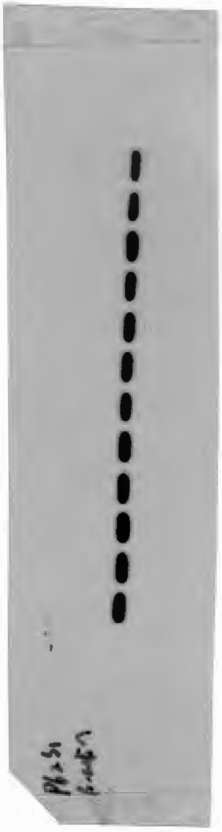

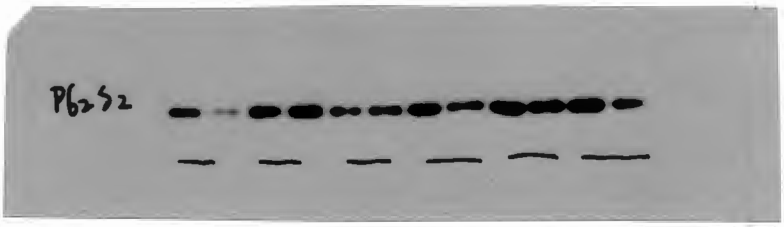
Pb2S2

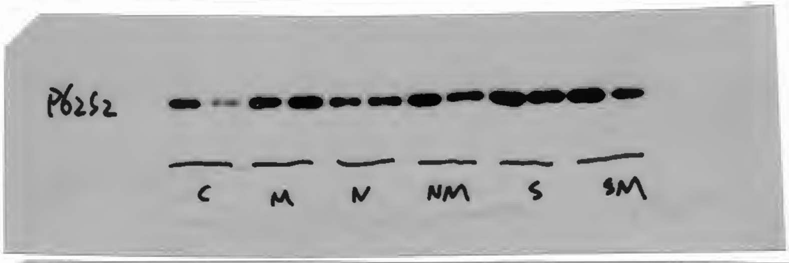
Pb2S2
C M N NM S SM

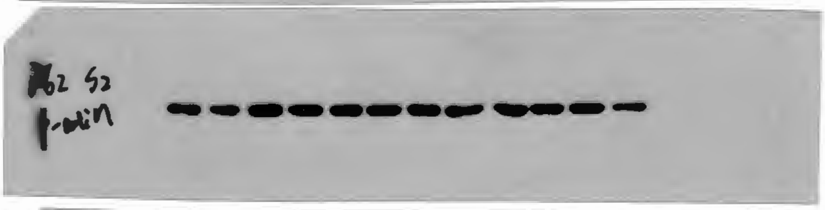
Pb2 S2
β-actin

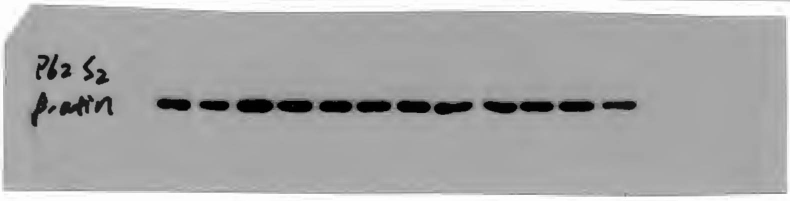
Pb2 S2
β-actin

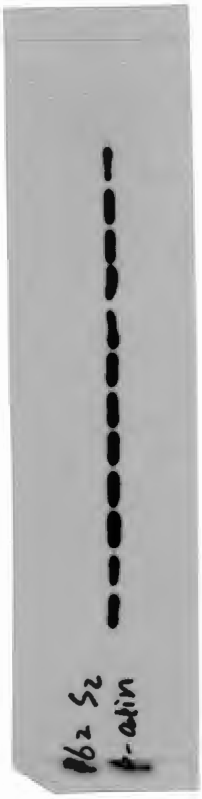

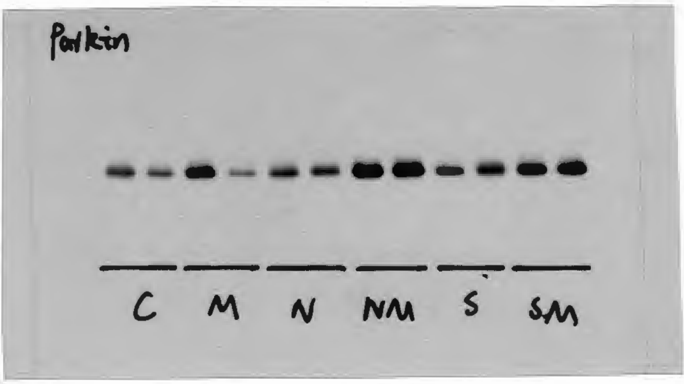
Parkin
C M N NM S SM

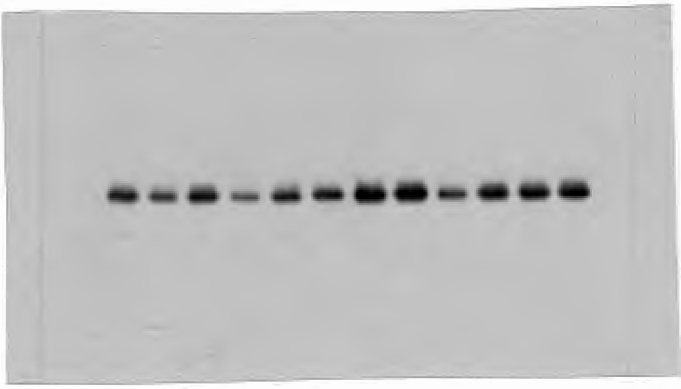

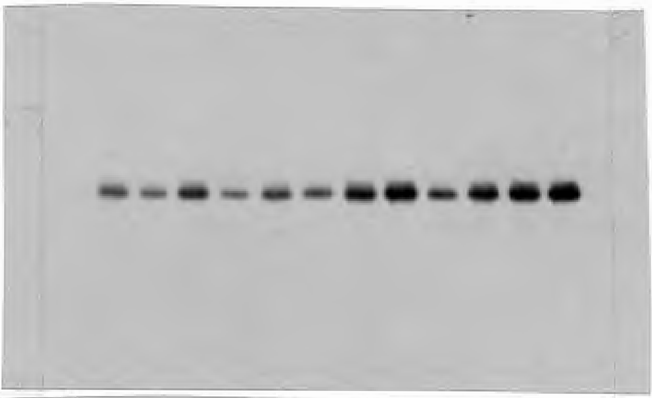

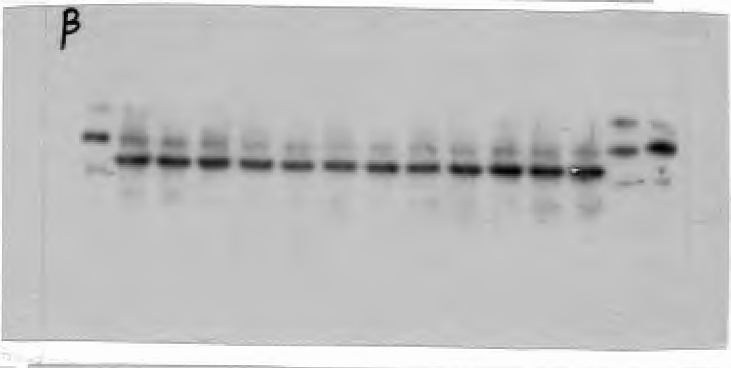
β

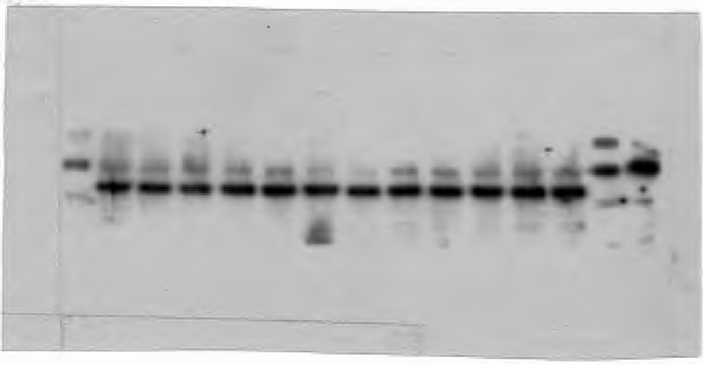

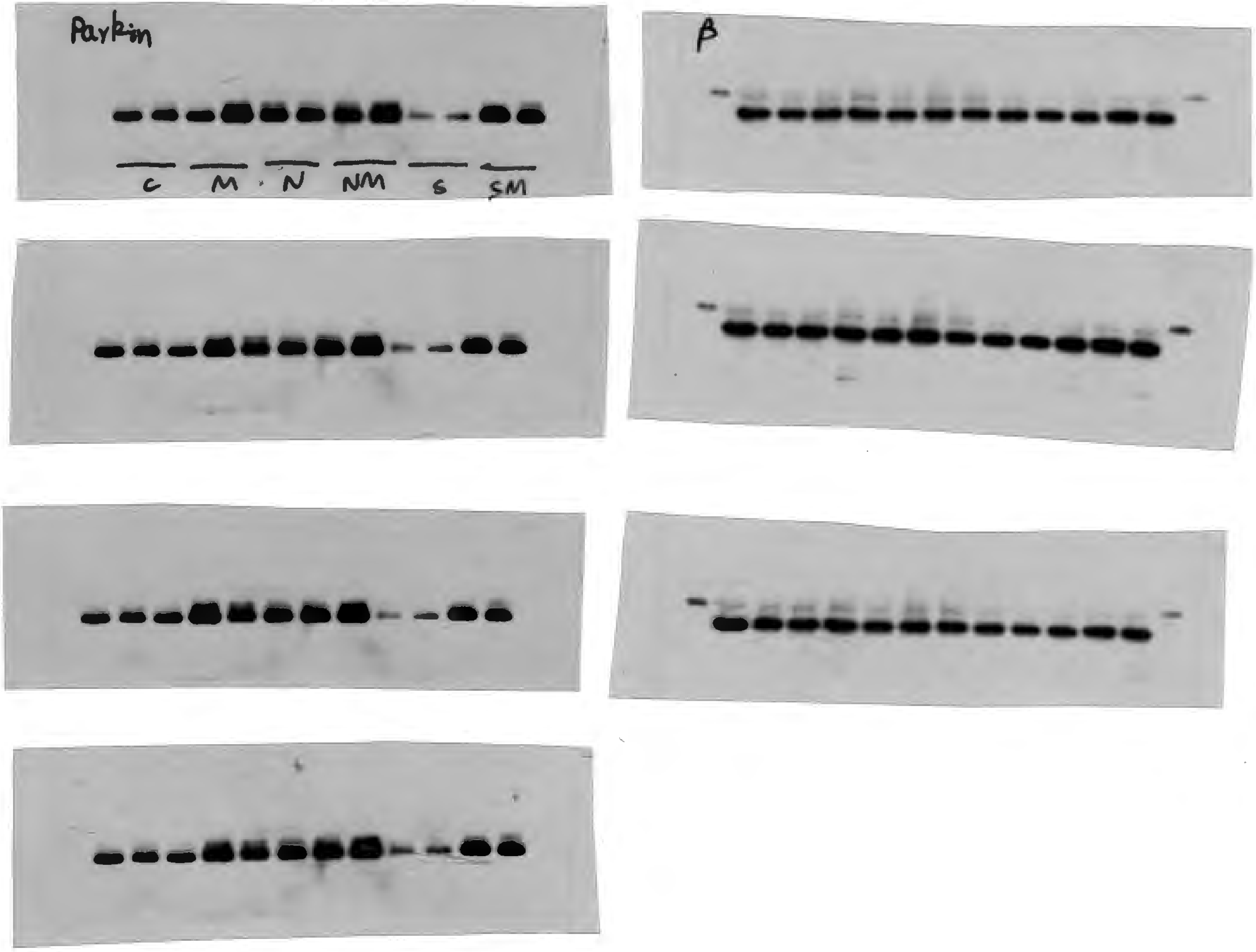
Parkin
C
M
N
NM
S
SM
β

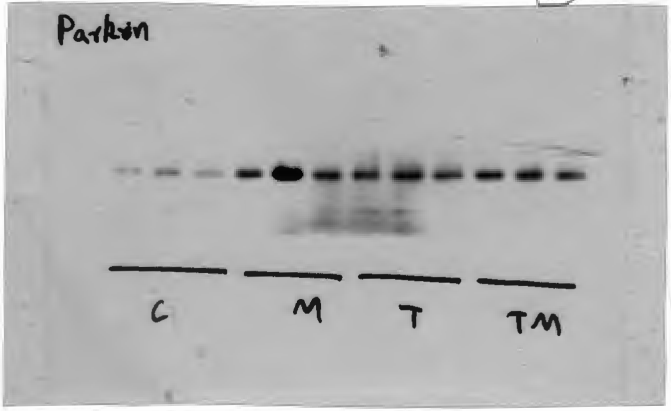
Parkin
C
M
T
TM

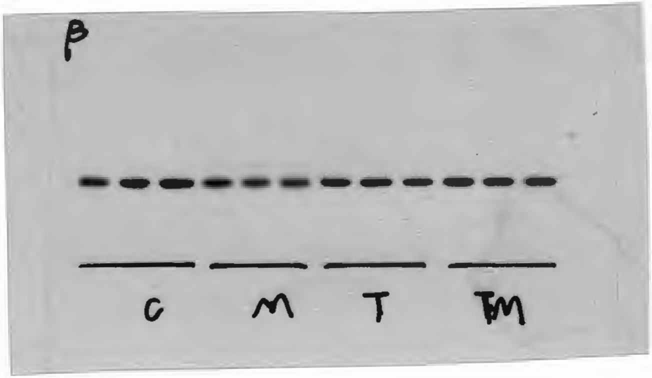
β
C
M
T
TM

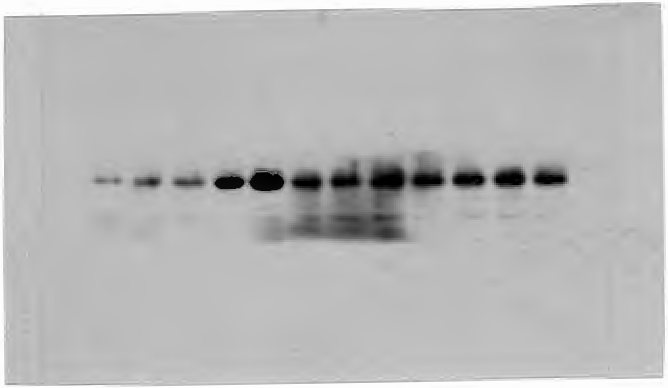

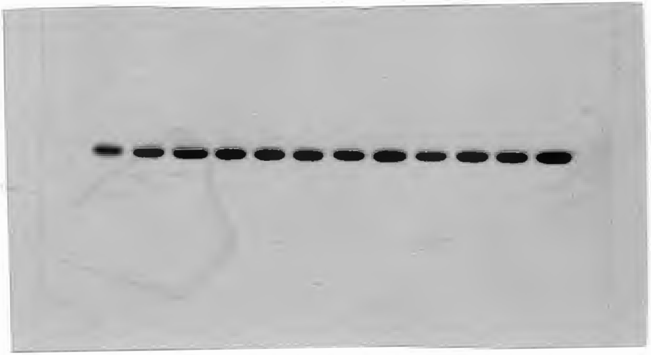

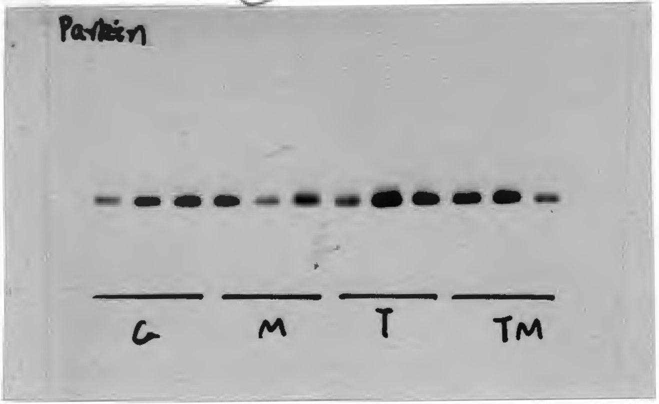
Parkin
G
M
T
TM

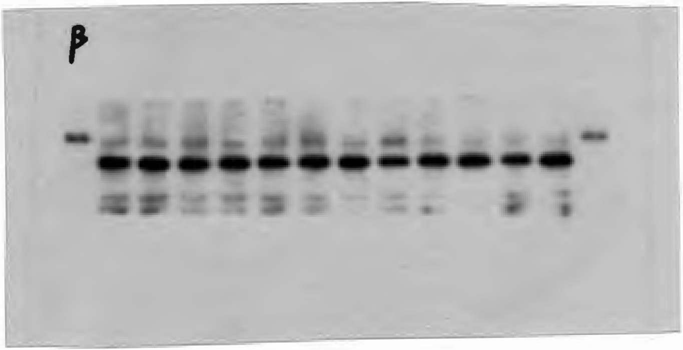
β

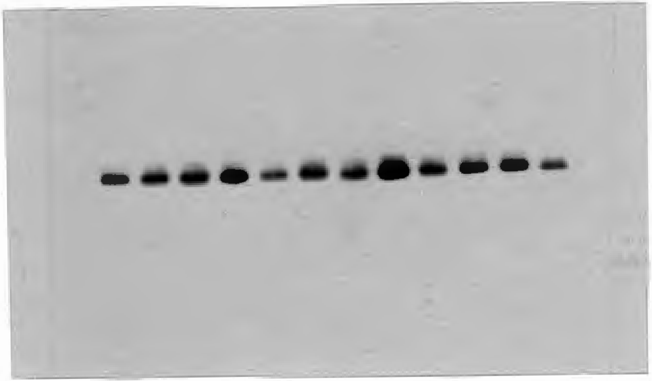

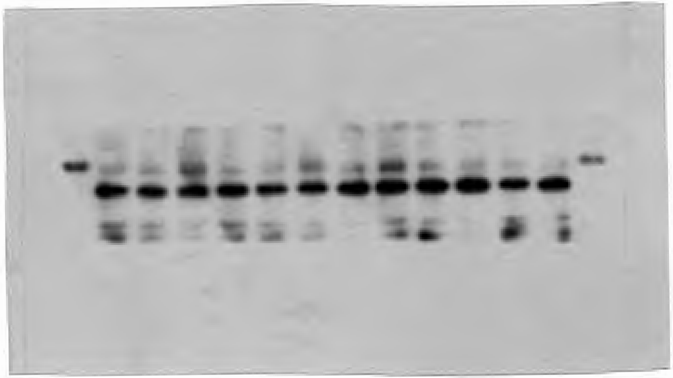

Trx-1 [illegible]. 2019 [illegible] SupC [illegible]

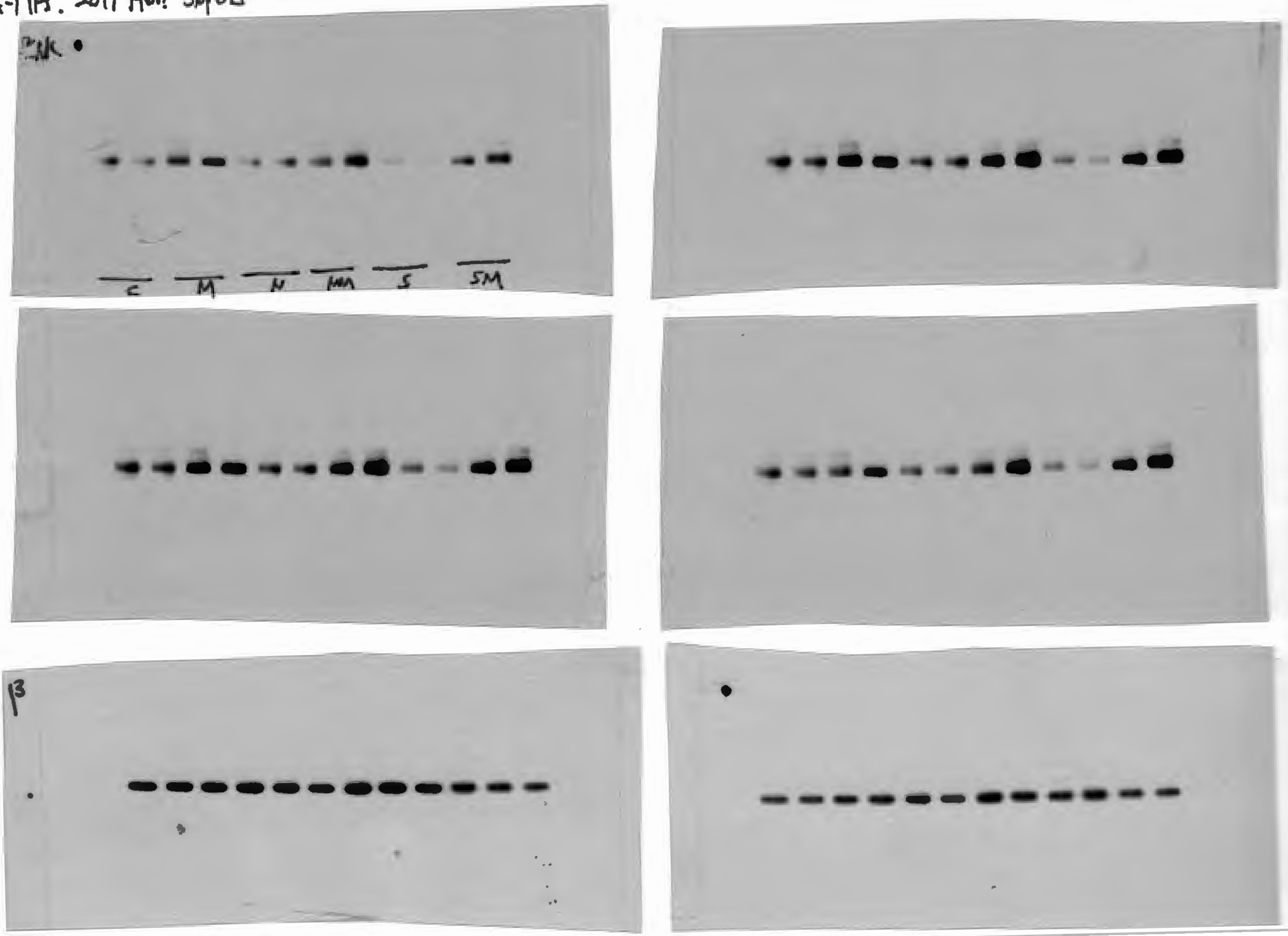

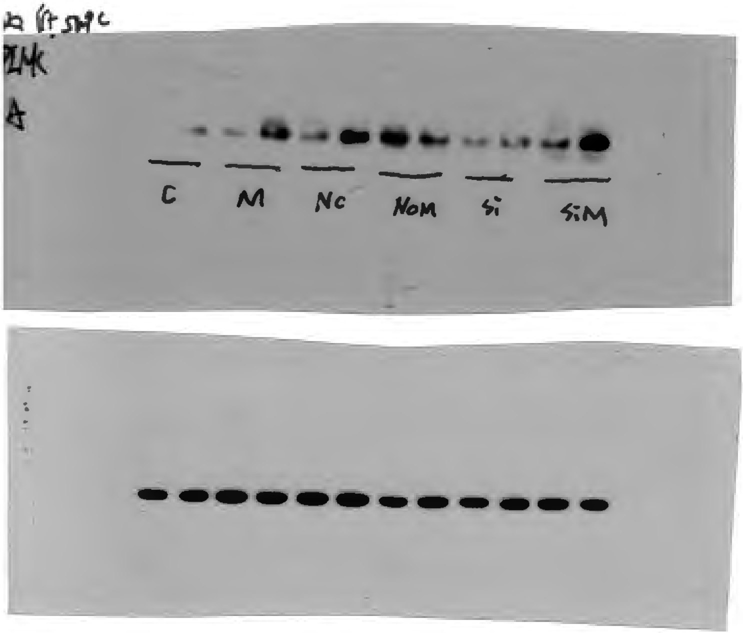
C
M
Nc
Nom
Si
SiM

2019.[illegible]

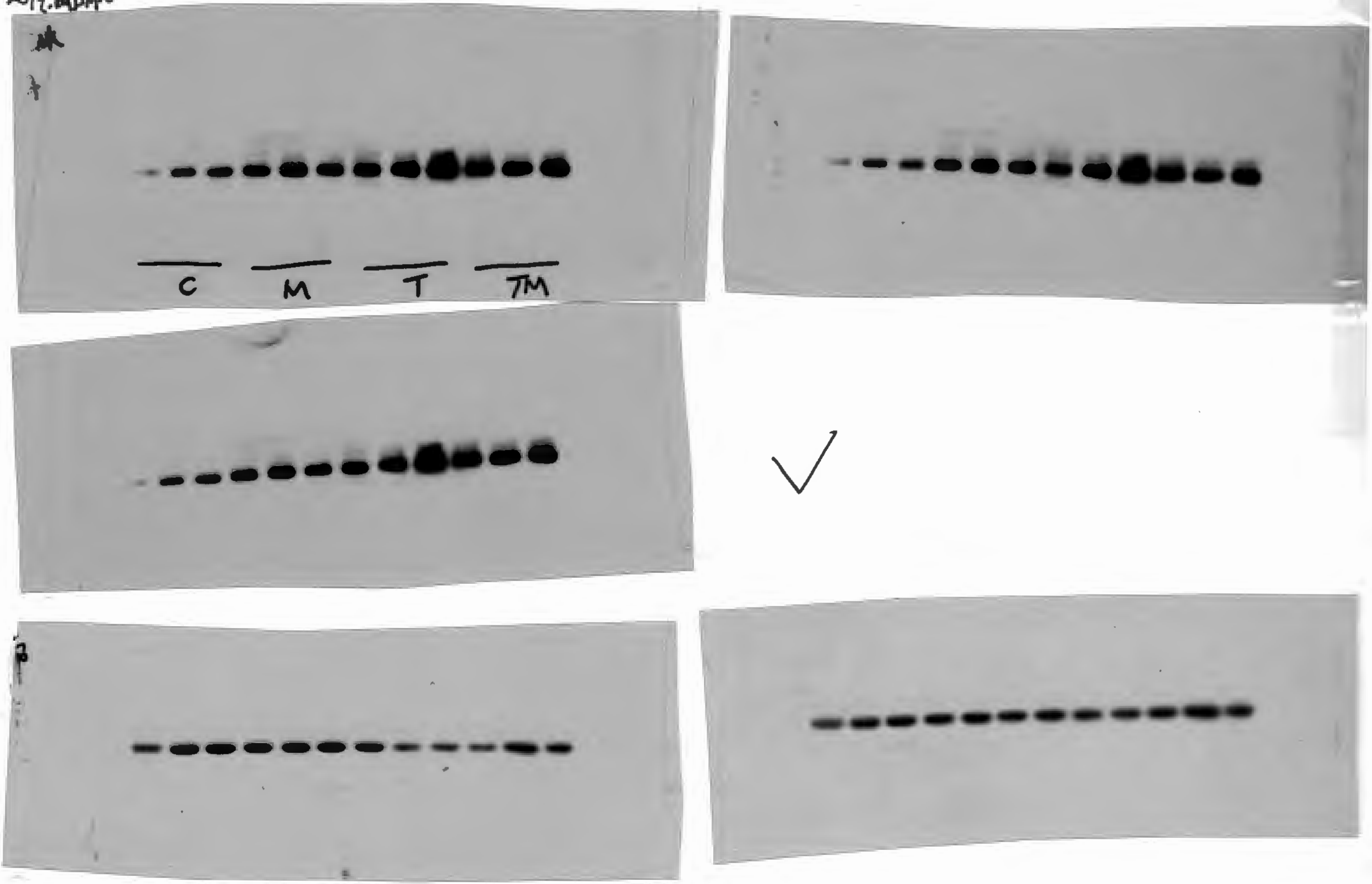

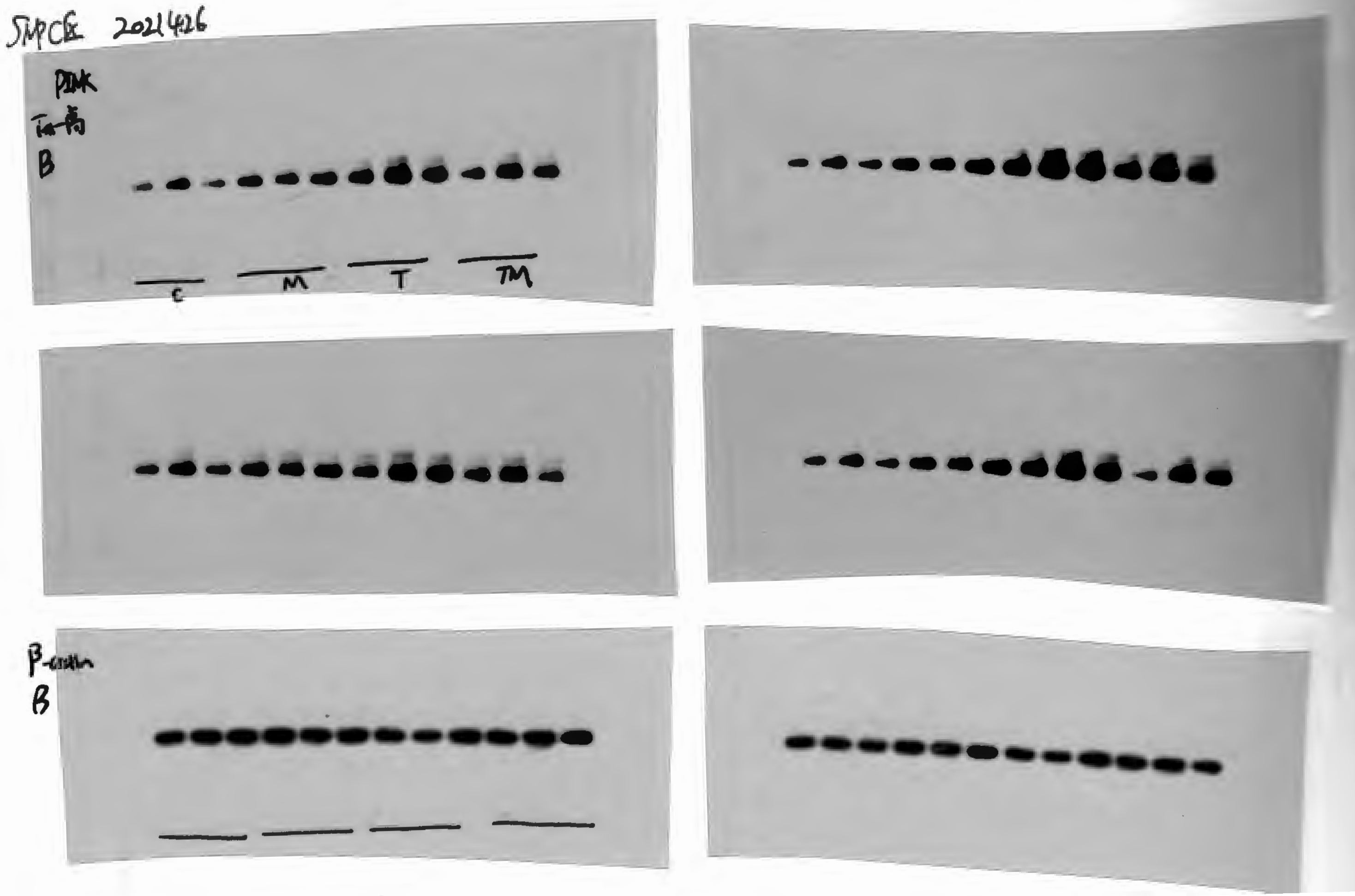
2021426
PINK
B
C
M
T
TM
β-actin
B

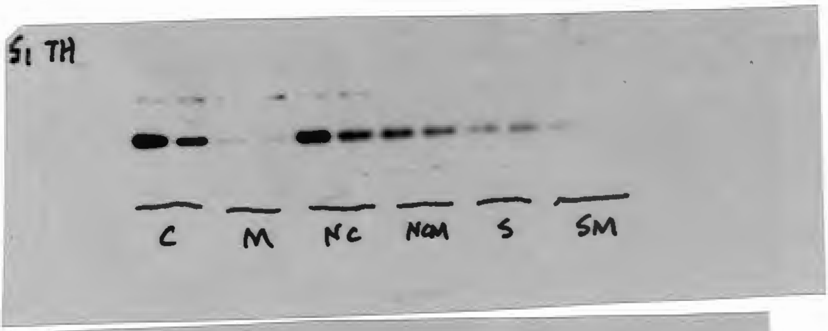
S1 TH
C
M
NC
NCM
S
SM

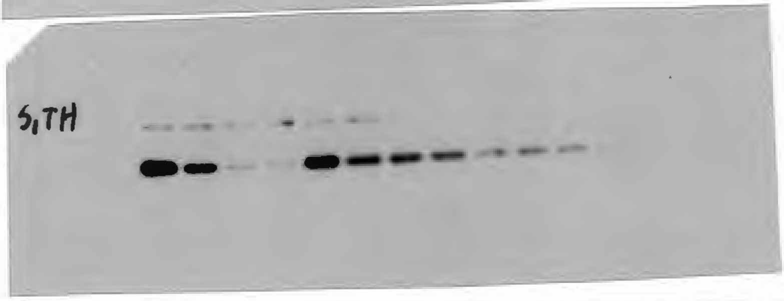
S1 TH

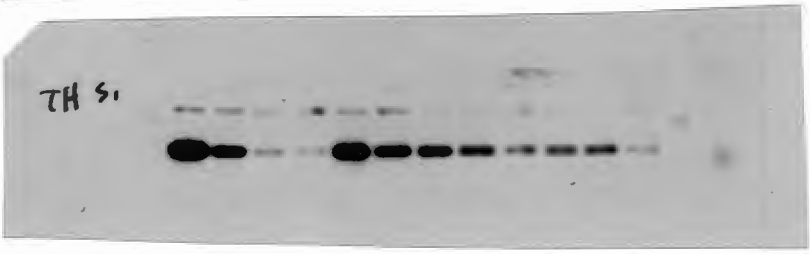
TH S1

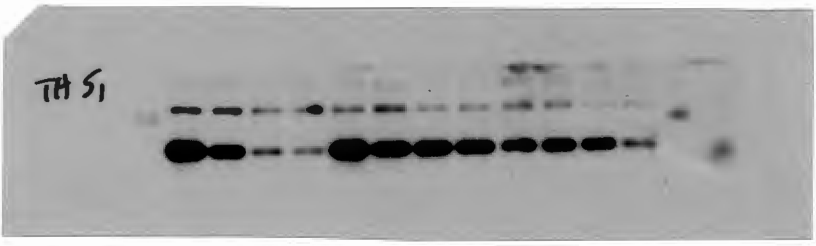
TH S1

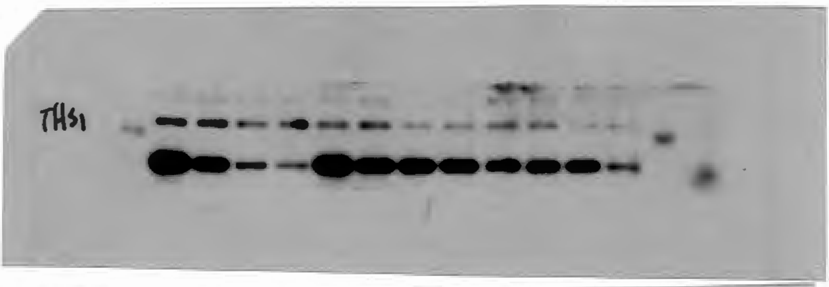
THS1

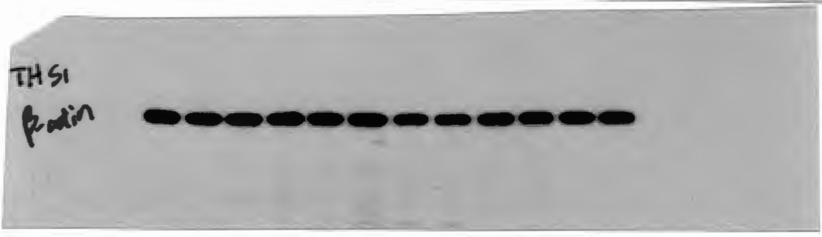
TH S1
β-actin

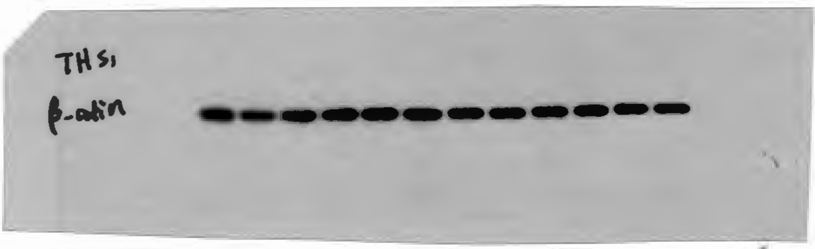
TH S1
β-actin

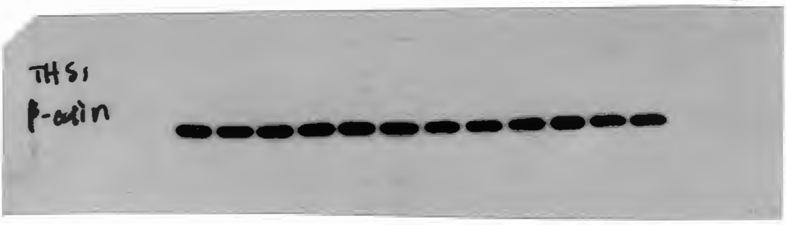
TH S1
β-actin

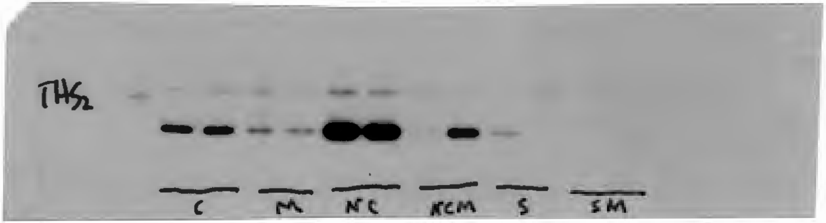
THS2
C
M
NC
KCM
S
SM

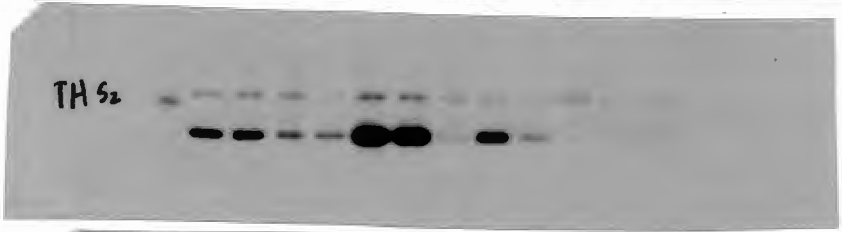
THS2

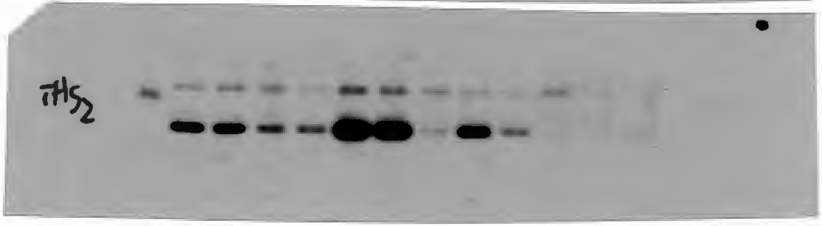
THS2

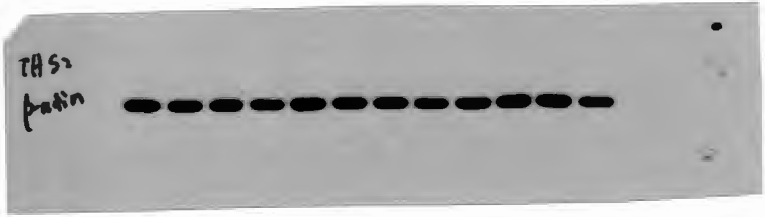
THS2

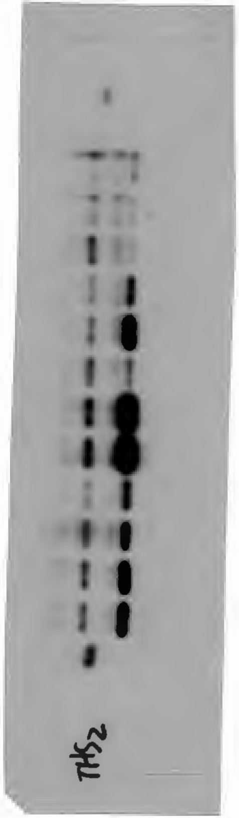
THS2

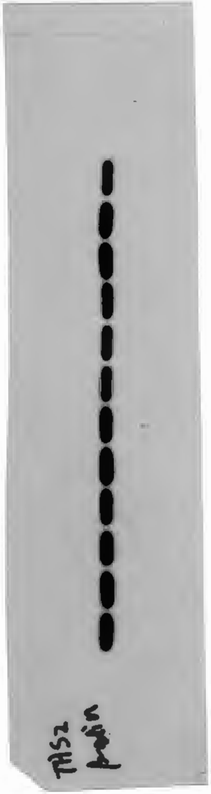
THS2

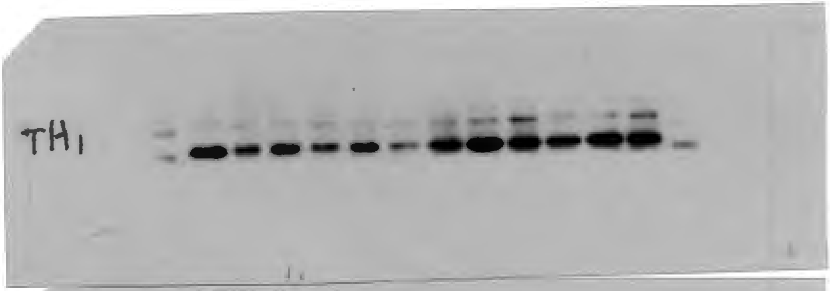
TH1
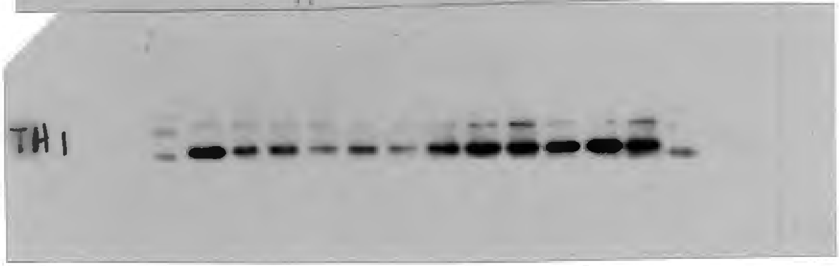
TH1
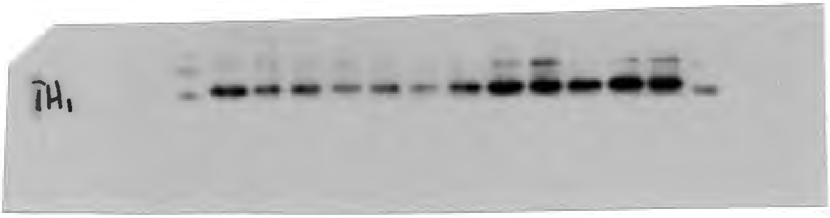
TH1
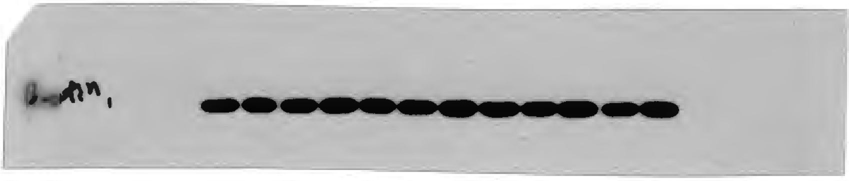
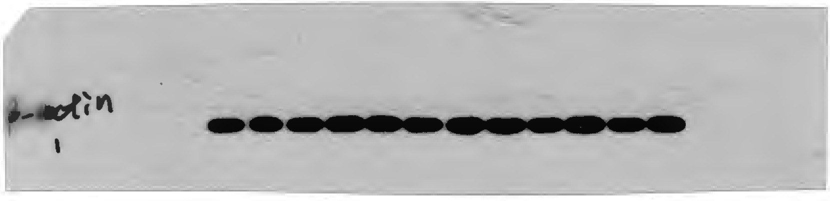
β-actin

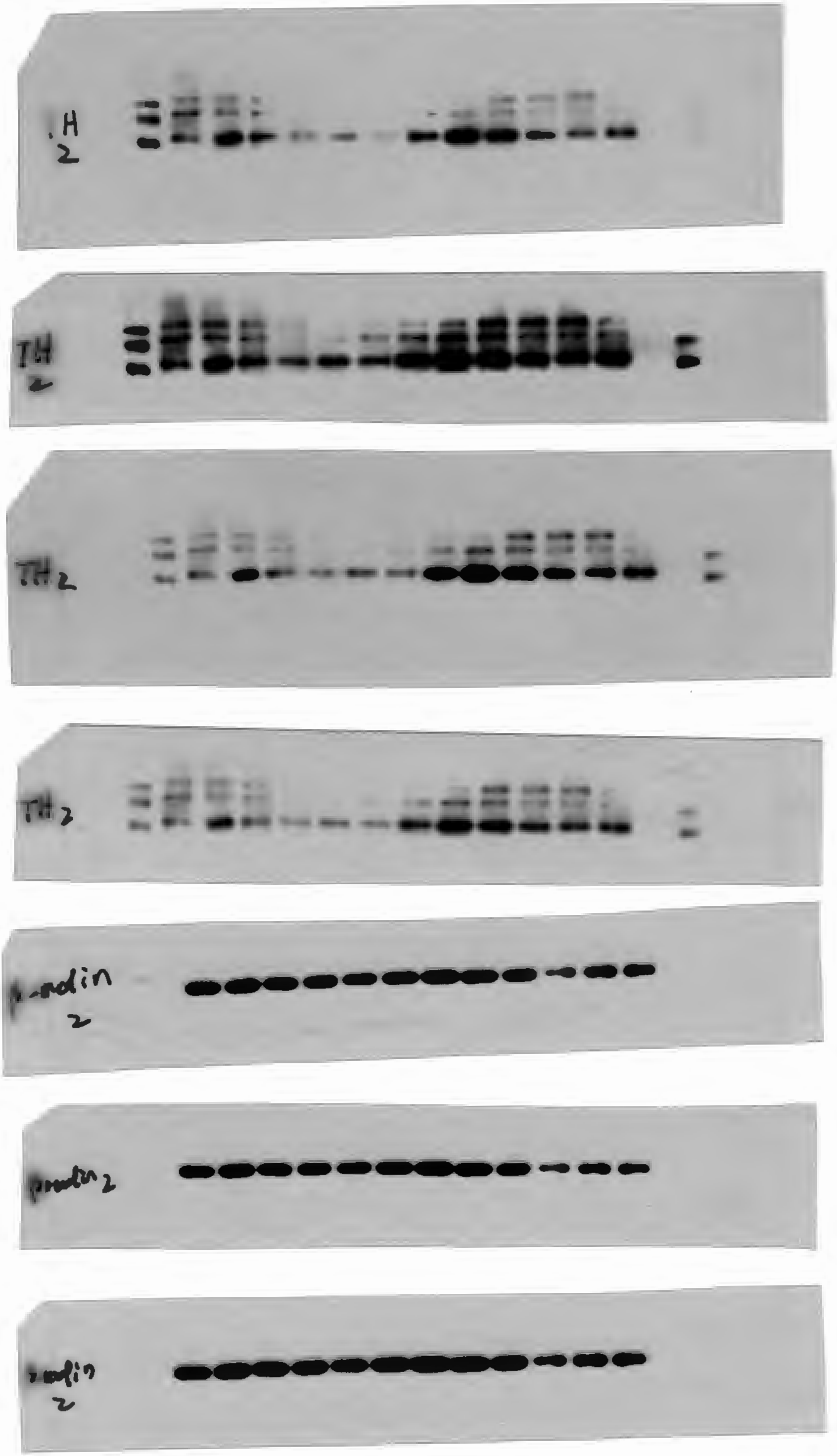
TH 2
TH 2
TH 2

PD 低表达

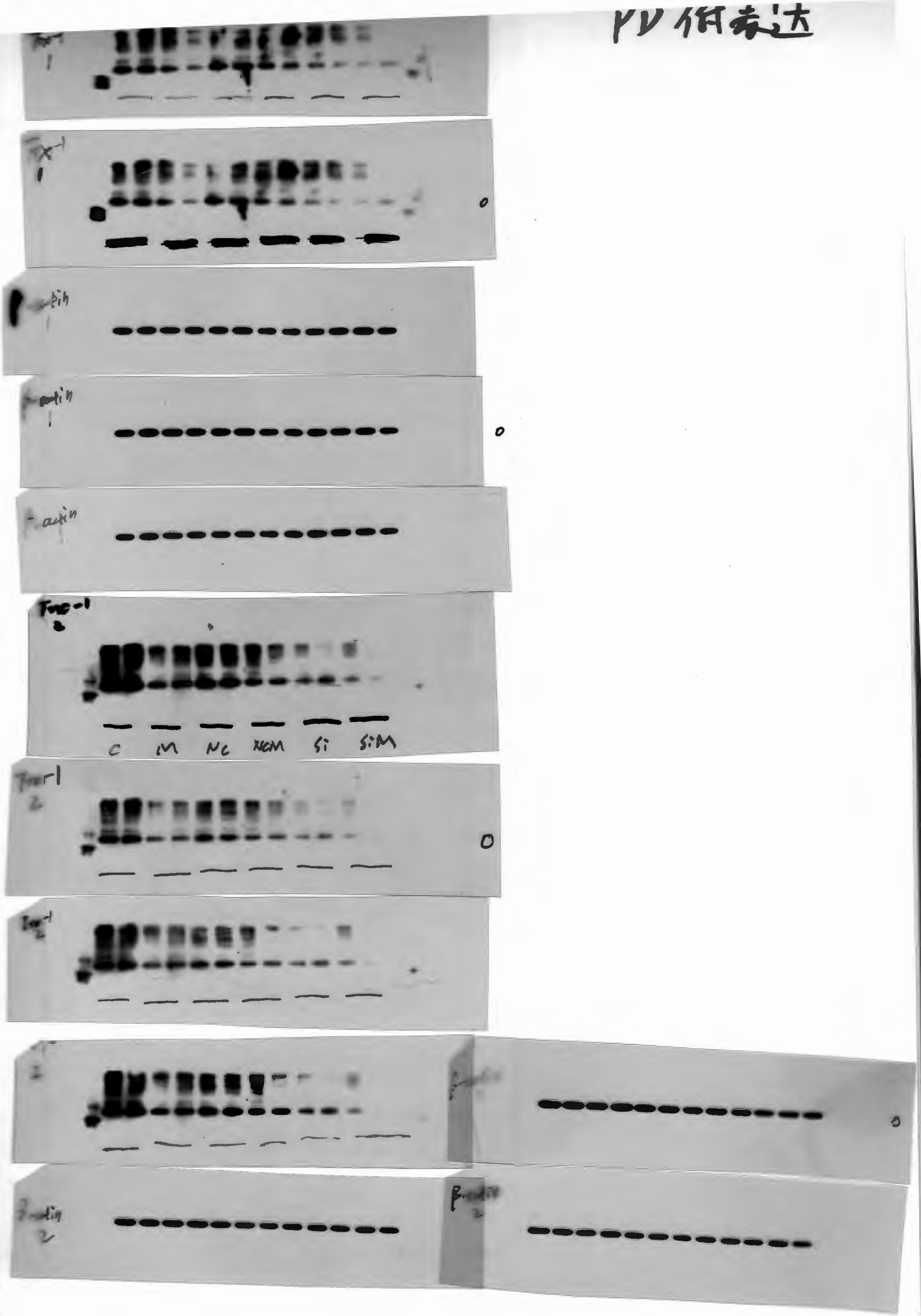

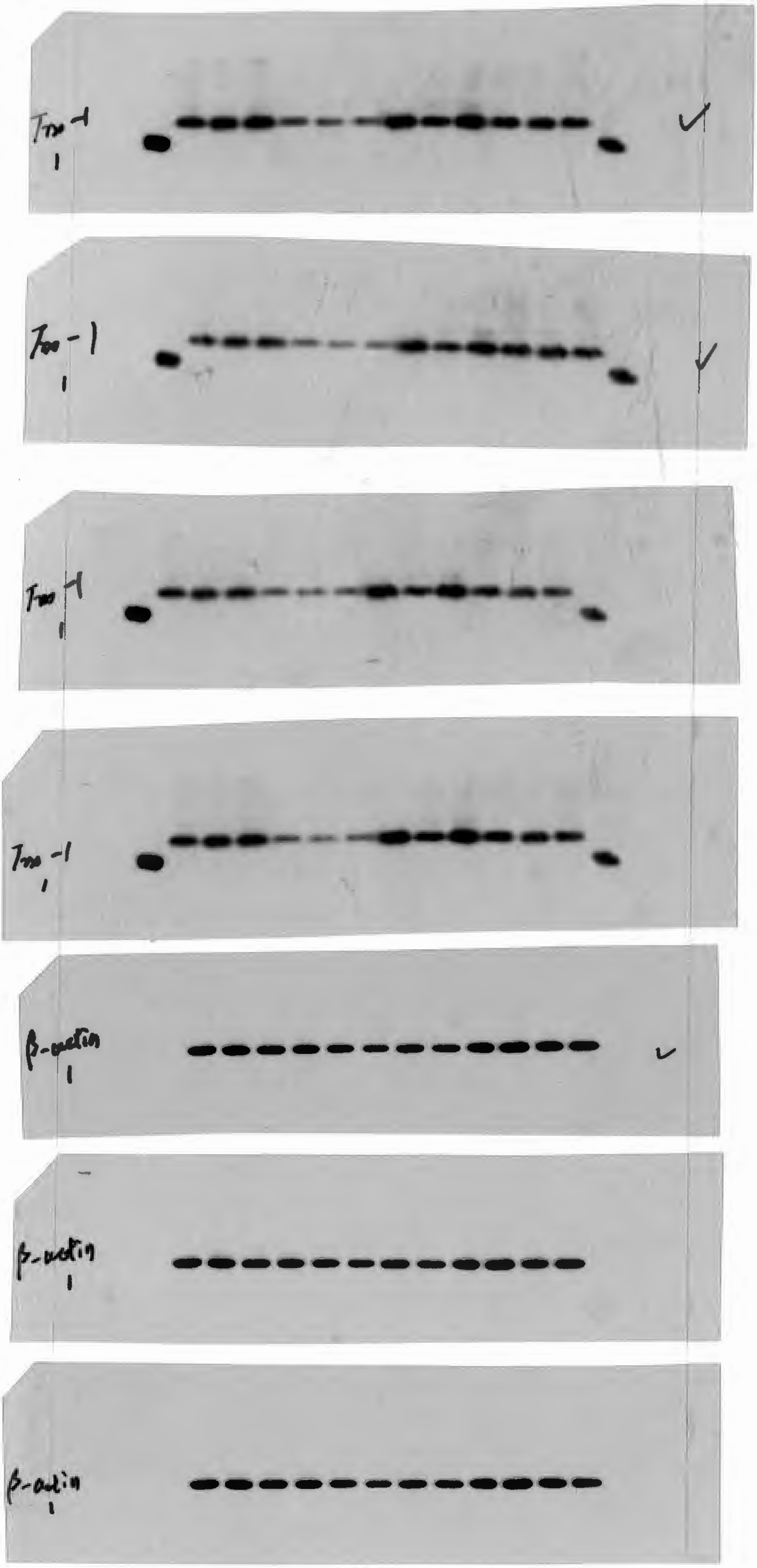
β-actin
β-actin
β-actin

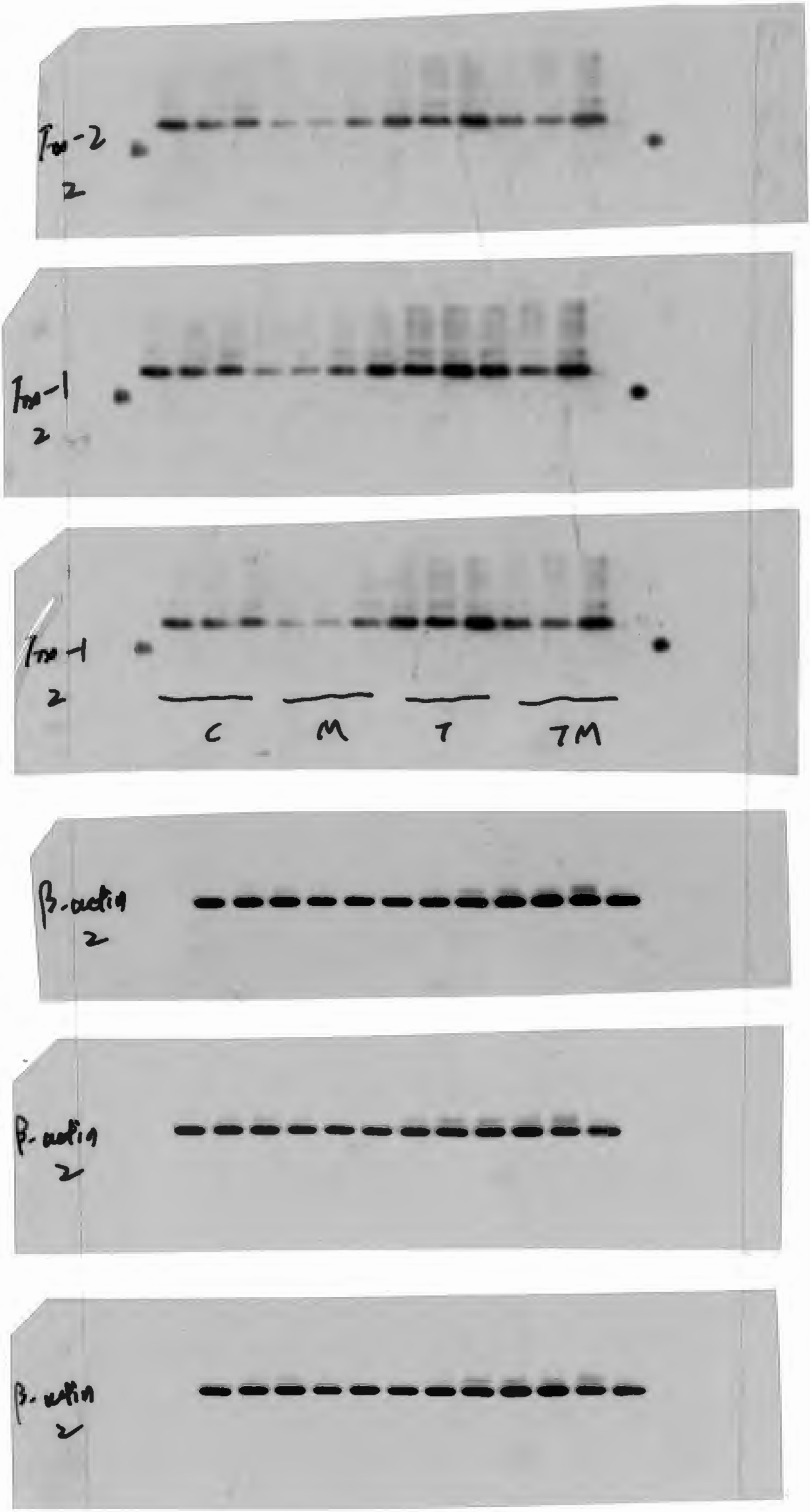
Trx-2
2
Trx-1
2
Trx-1
2
C
M
T
TM
β-actin
2
β-actin
2
β-actin
2

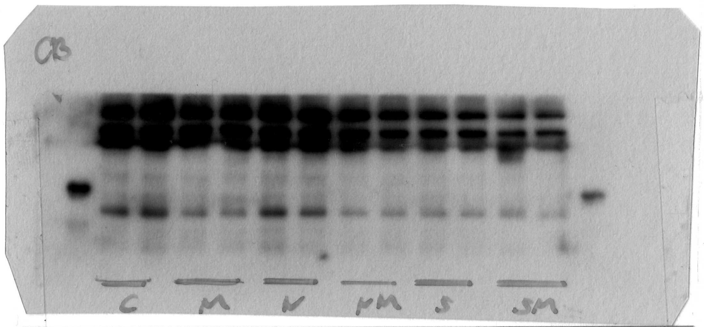
C
M
S
SM

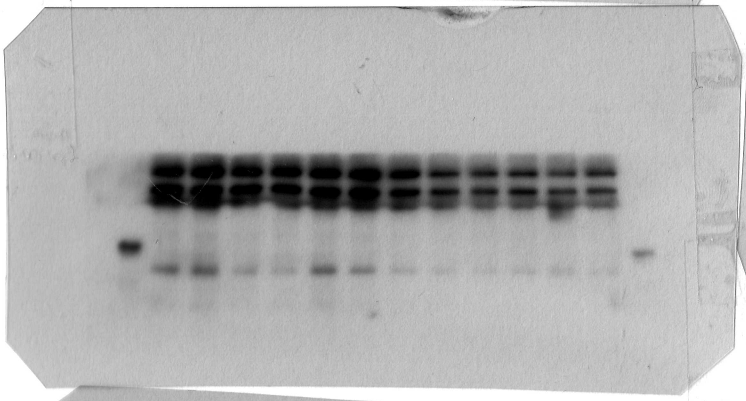

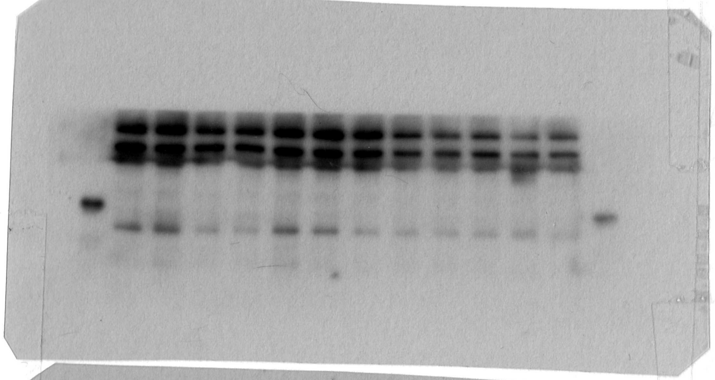

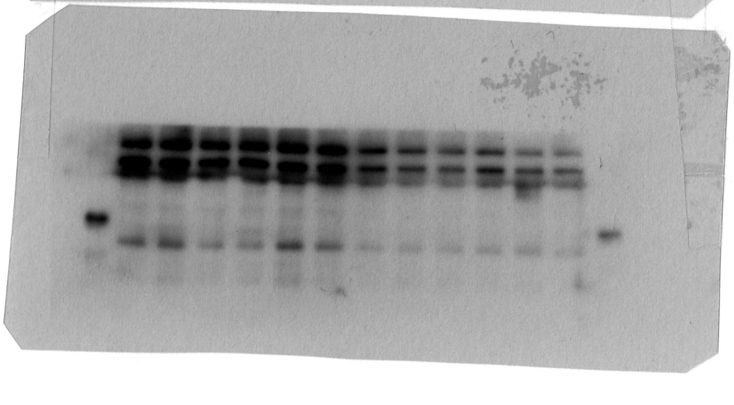

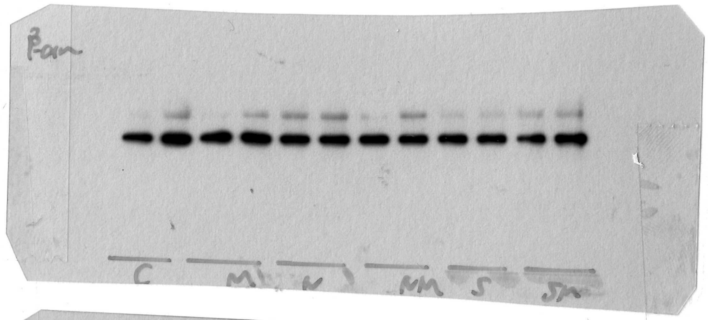
C
M
S

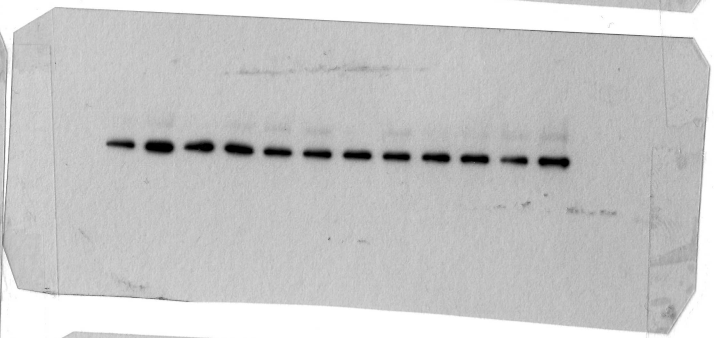

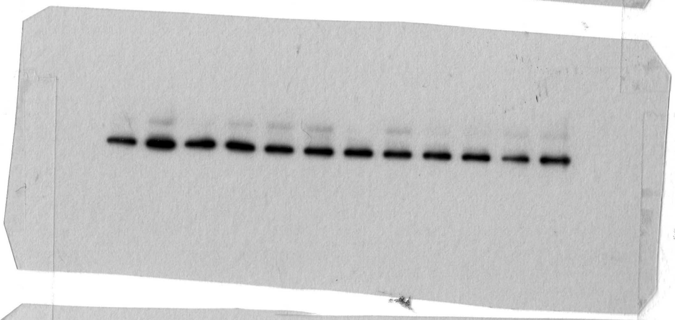

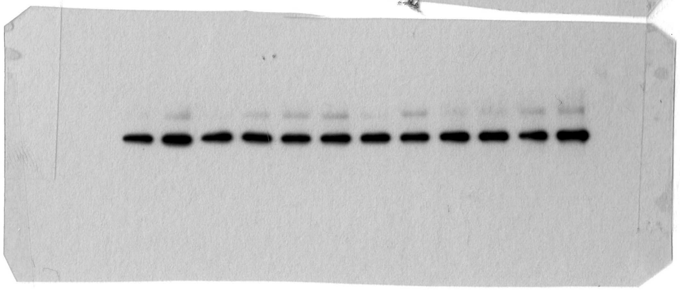

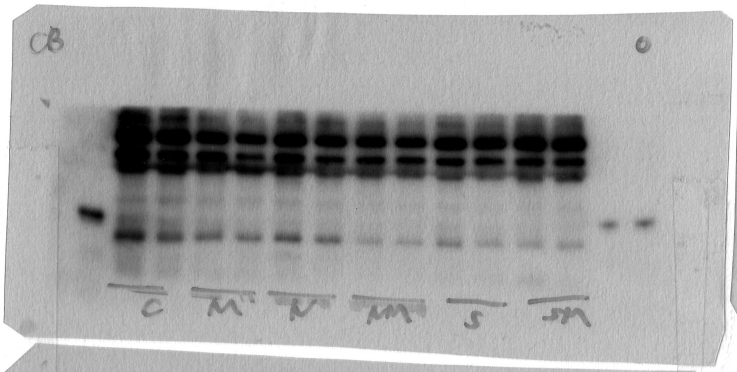
CB
C M N MM S SM

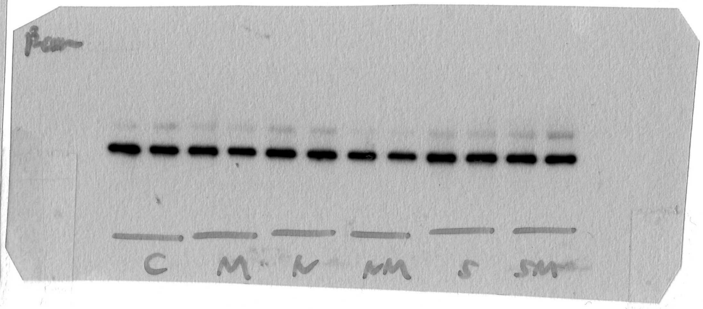
C M N MM S SM

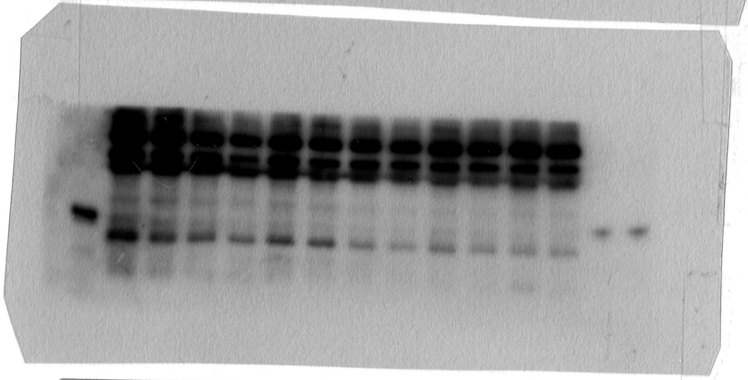

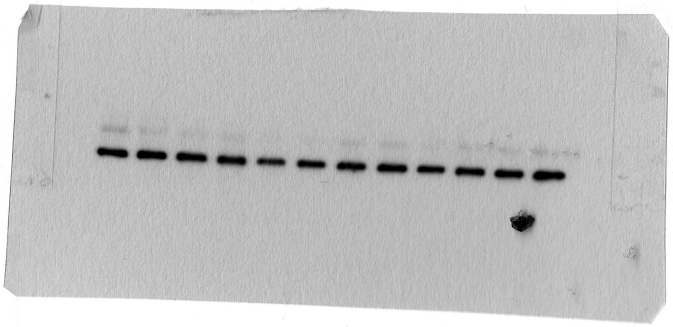

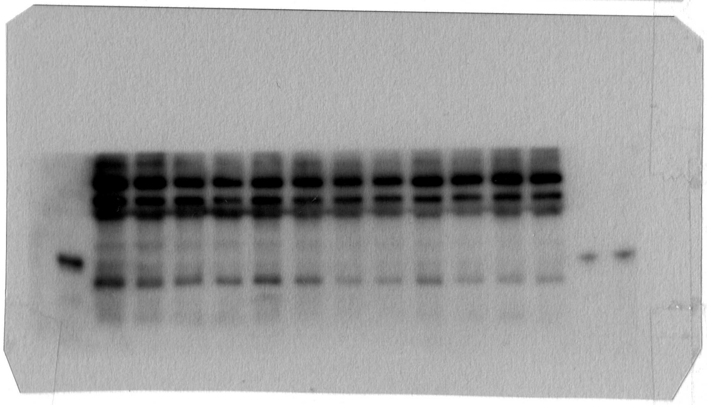

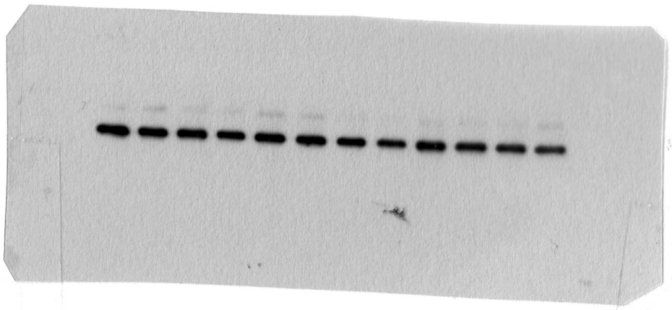

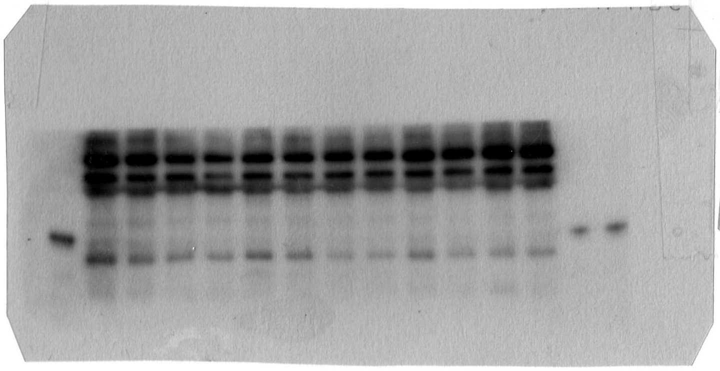

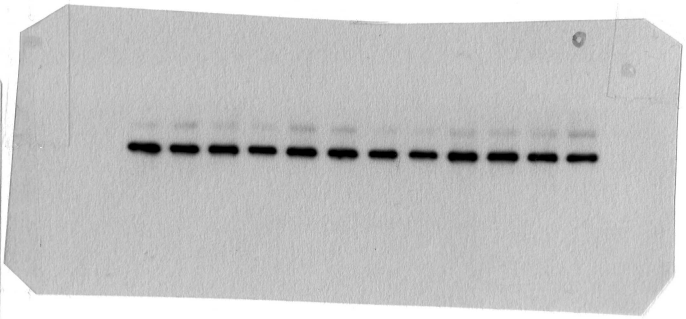

Trx高表达 CathepsinB SrvpC 2023.12.8

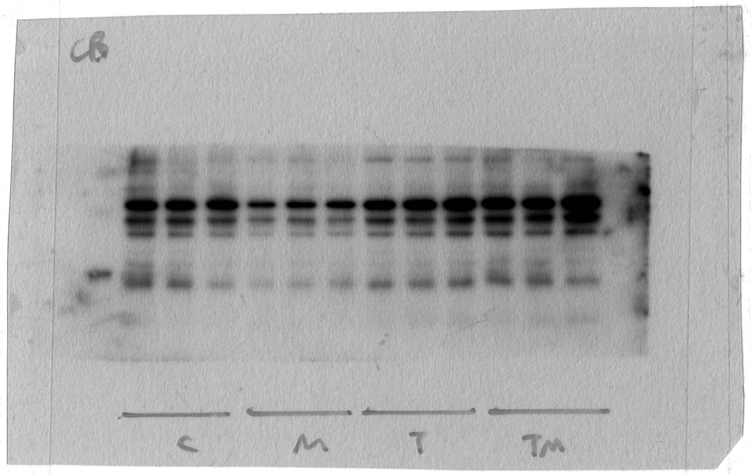

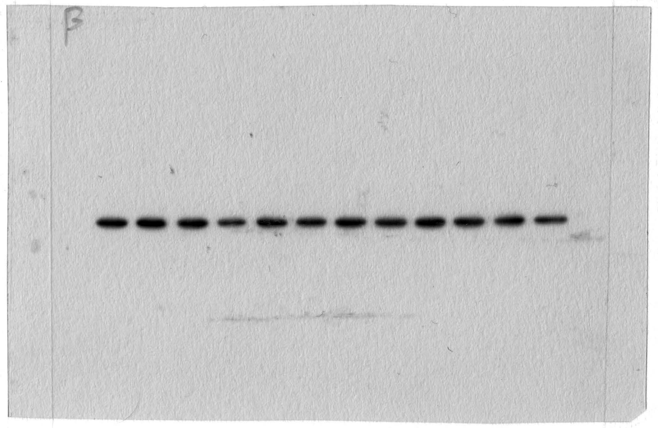

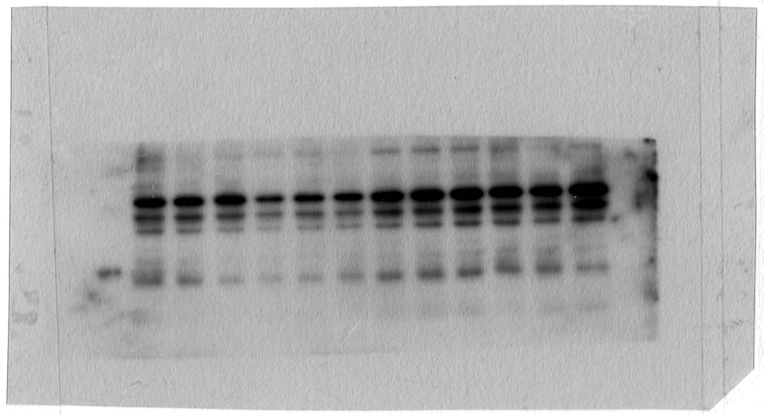

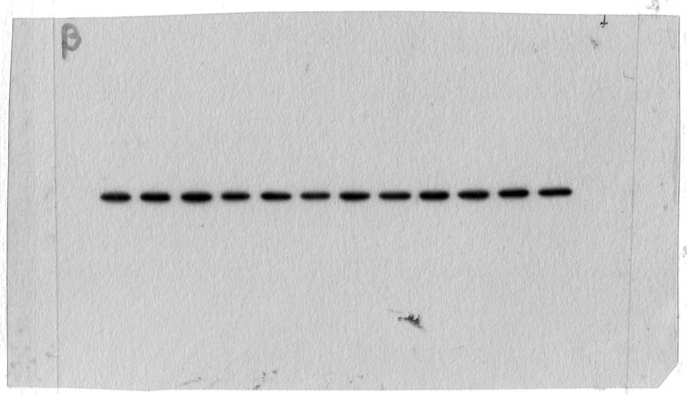

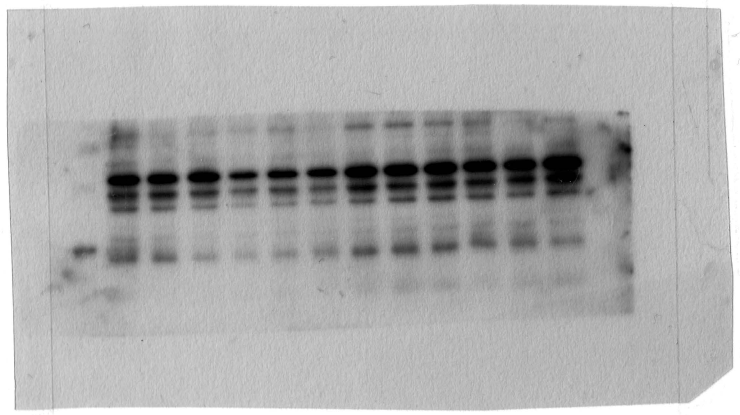

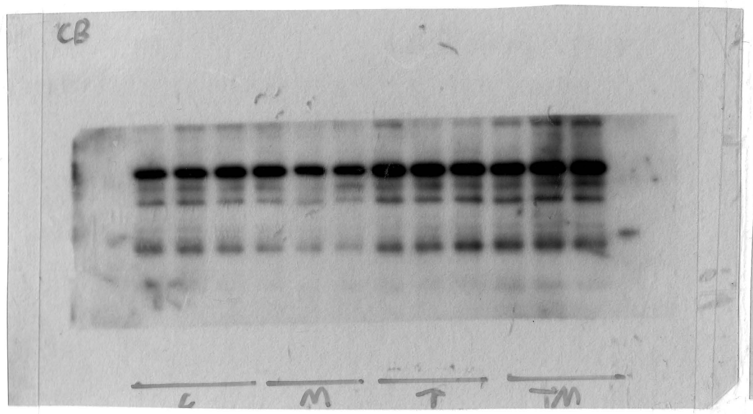

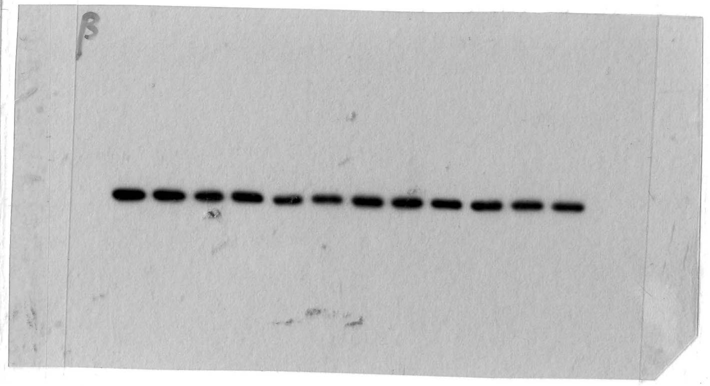

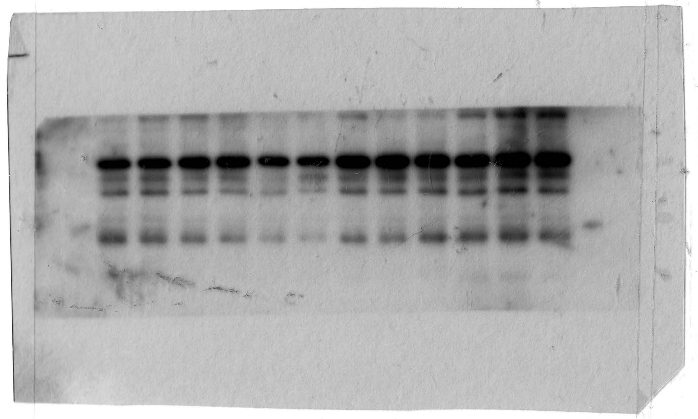

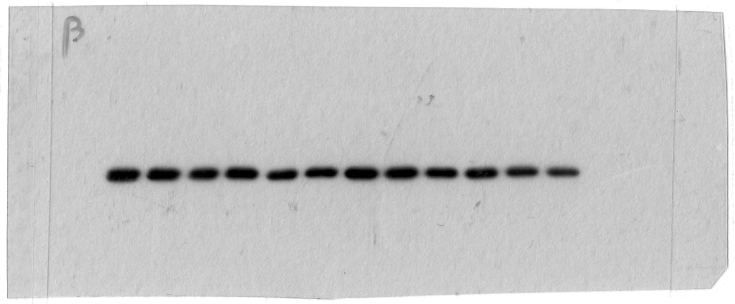

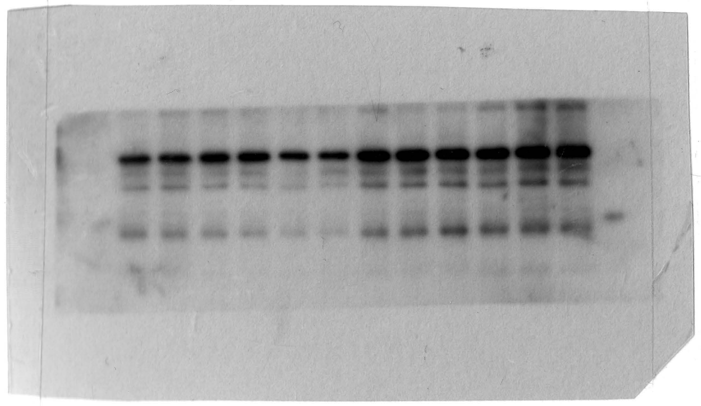

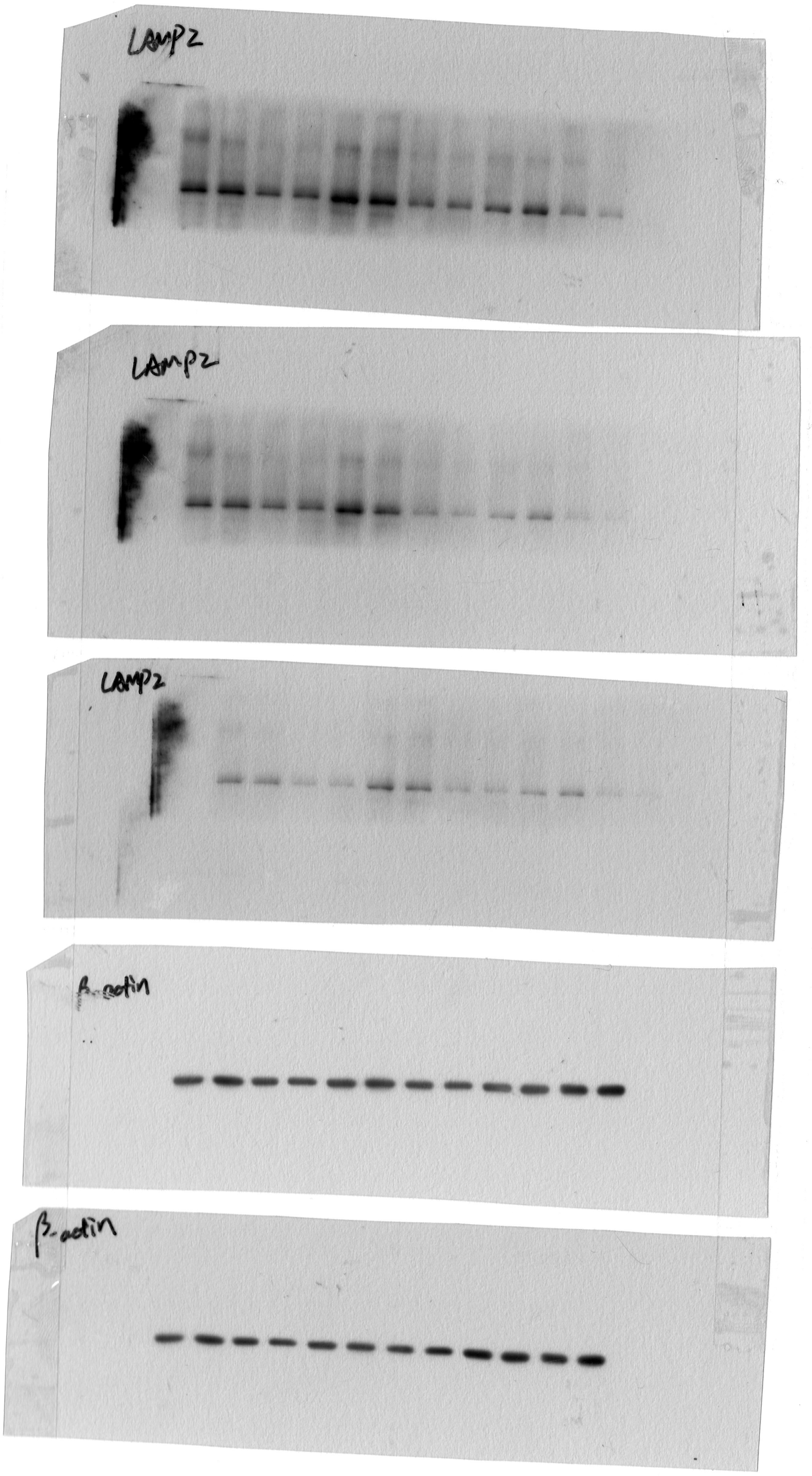
LAMP2
LAMP2
LAMP2
β-actin
β-actin

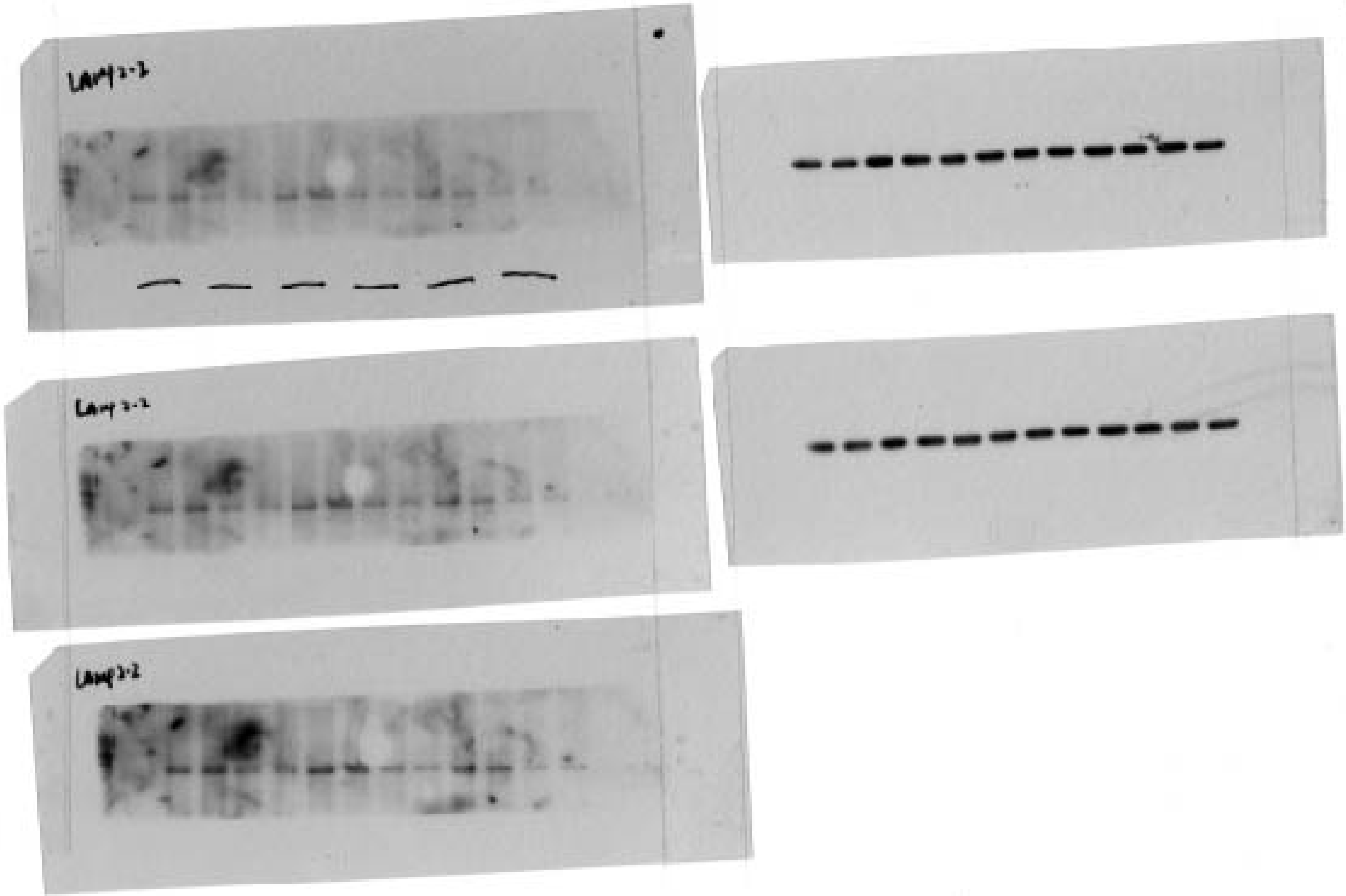

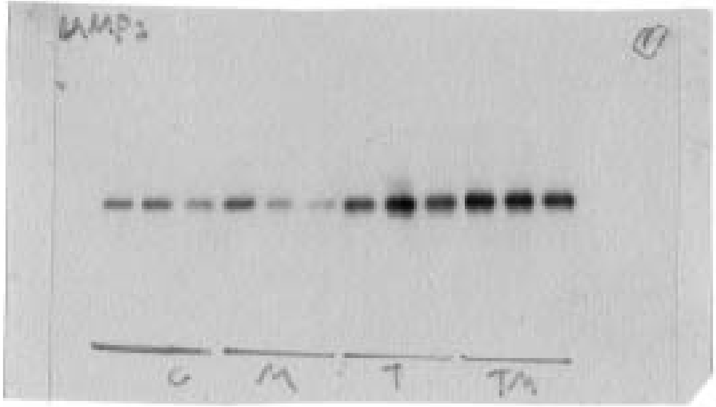
LAMP2
C
M
T
TM

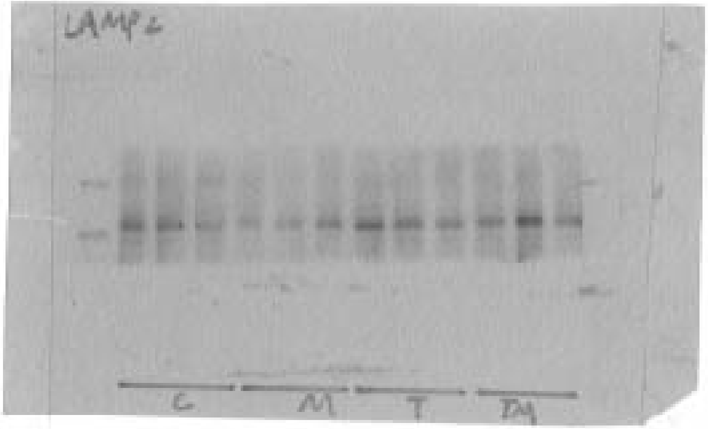
LAMP2
C
M
T
TM

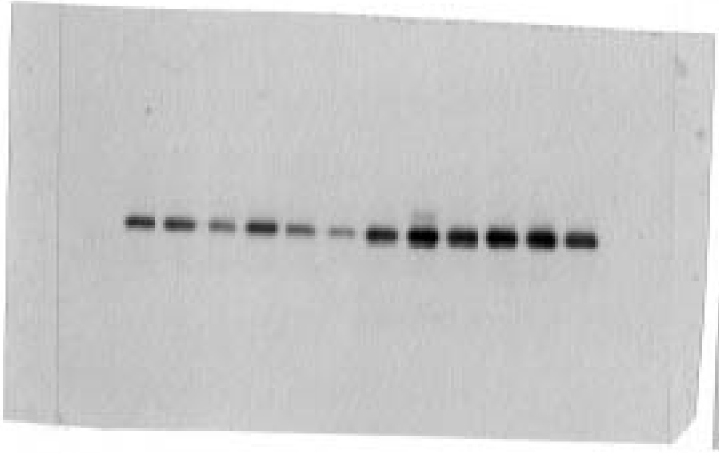

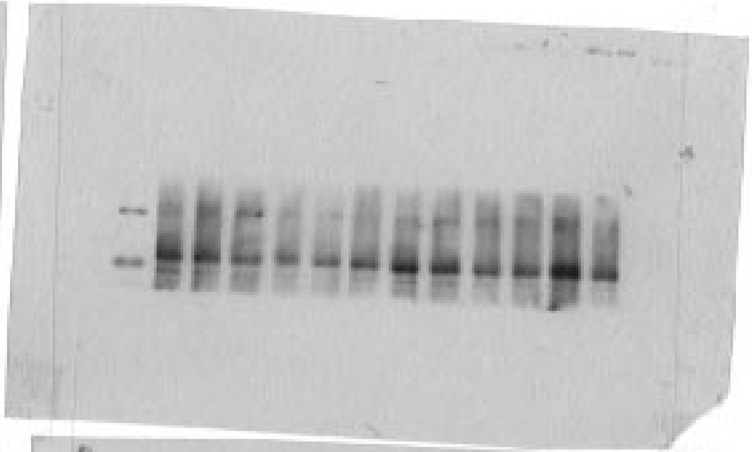

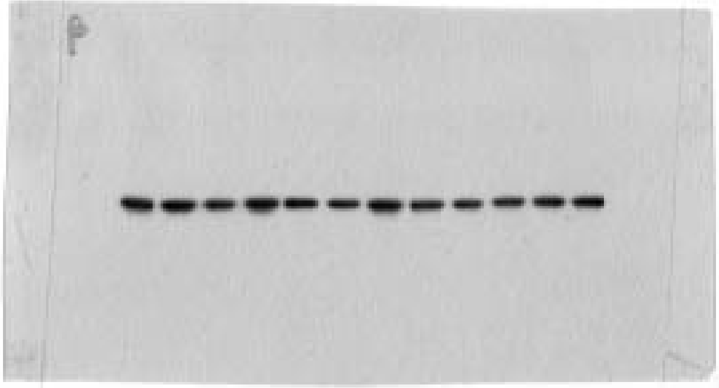
β

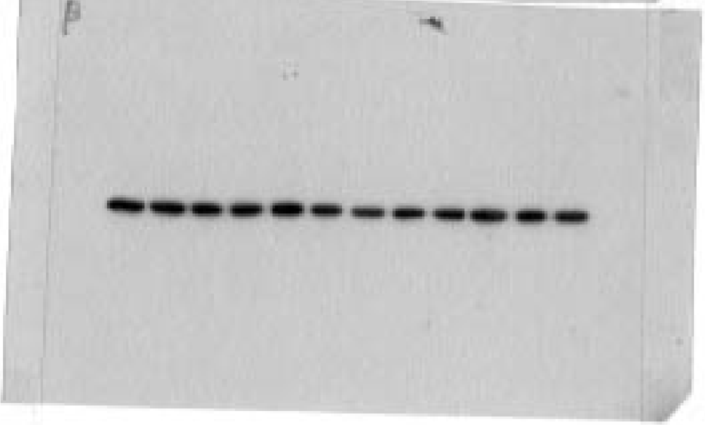
β

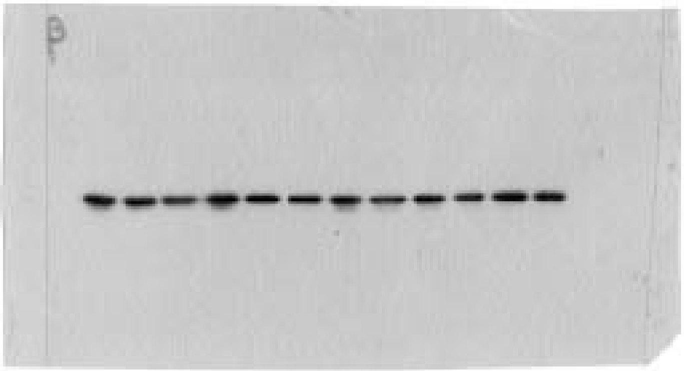
β

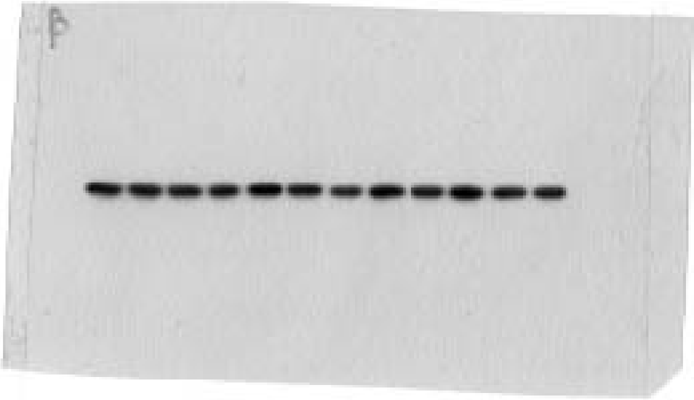
β

2021.10.19. Trx 低表达模型 SNpc

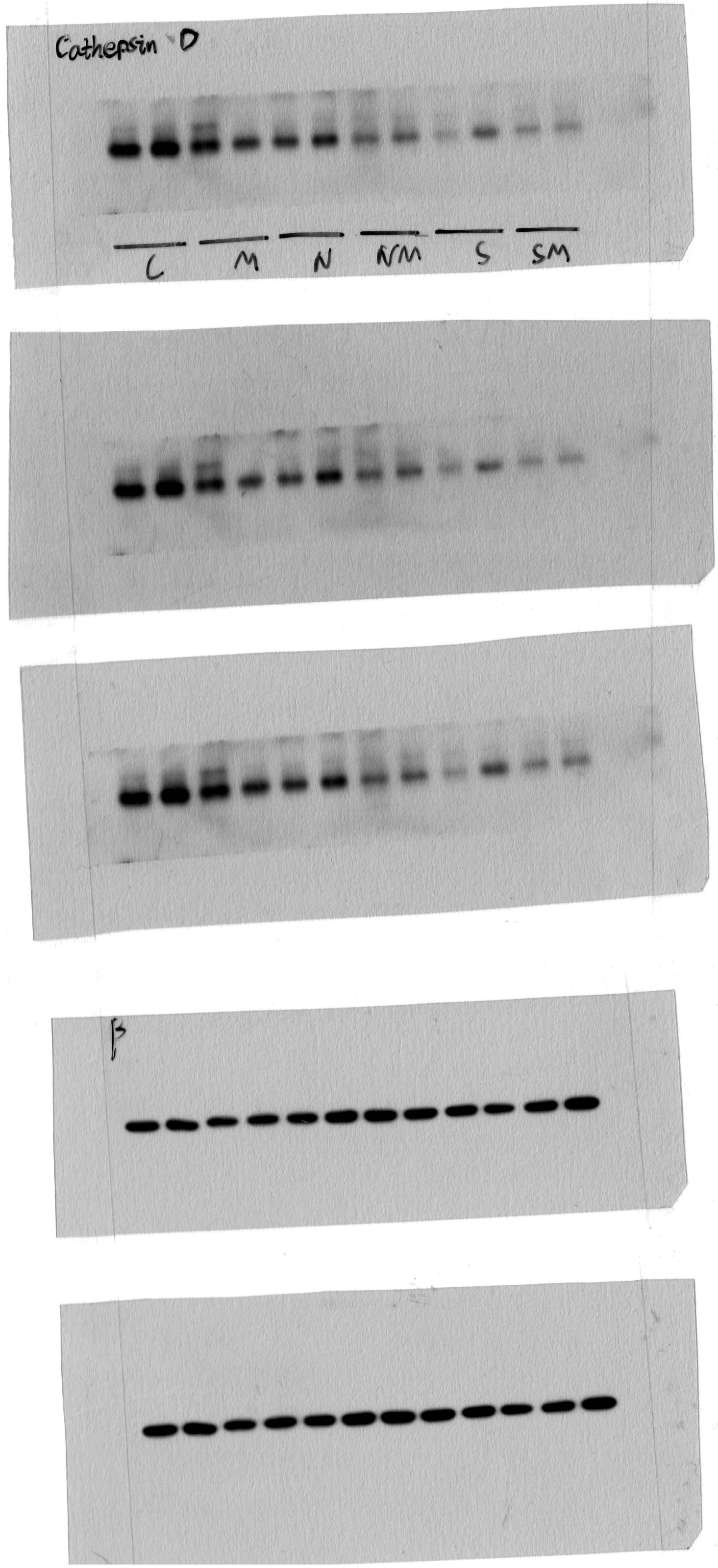

Supplement: Supplementary file 1 — Supplementary materials [file 41420_2024_1848_MOESM1_ESM.pdf]
